# Occurrence of “Natural Selection” in Successful Small Molecule Drug Discovery

**DOI:** 10.1021/acs.jmedchem.4c00811

**Published:** 2024-07-01

**Authors:** A. Lina Heinzke, Axel Pahl, Barbara Zdrazil, Andrew R. Leach, Herbert Waldmann, Robert J. Young, Paul D. Leeson

**Affiliations:** † European Molecular Biology Laboratory, European Bioinformatics Institute, Wellcome Genome Campus, Hinxton CB10 1SD, Cambridgeshire, U.K.; ‡ Compound Management and Screening Center, Max-Planck-Institute of Molecular Physiology, Otto-Hahn-Straße 11, 44227 Dortmund, Germany; § Department of Chemical Biology, Max-Planck-Institute of Molecular Physiology, Otto-Hahn-Straße 11, 44227 Dortmund, Germany; ∥ Faculty of Chemistry and Chemical Biology, Technical University Dortmund, Otto-Hahn-Straße 6, 44227 Dortmund, Germany; ⊥ Blue Burgundy Ltd., Ampthill, Bedfordshire MK45 2AD, U.K.; # Paul Leeson Consulting Ltd., Nuneaton CV13 6LZ, Warwickshire, U.K.

## Abstract

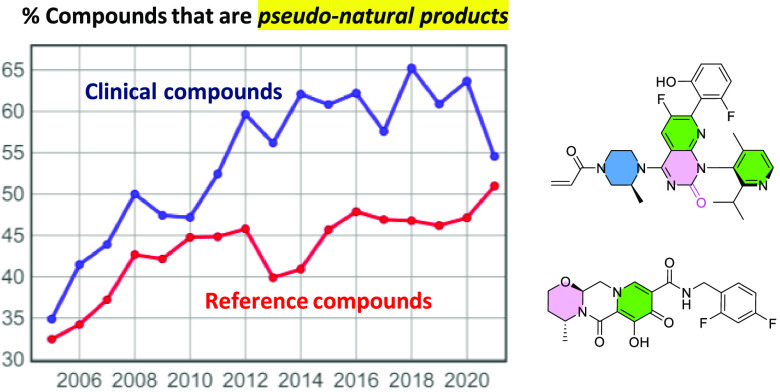

Published compounds from ChEMBL version 32 are used to seek evidence for the occurrence of “natural selection” in drug discovery. Three measures of natural product (NP) character were applied, to compare time- and target-matched compounds reaching the clinic (clinical compounds in phase 1–3 development and approved drugs) with background compounds (reference compounds). Pseudo-NPs (PNPs), containing NP fragments combined in ways inaccessible by nature, are increasing over time, reaching 67% of clinical compounds first disclosed since 2010. PNPs are 54% more likely to be found in post-2008 clinical versus reference compounds. The majority of target classes show increased clinical compound NP character versus their reference compounds. Only 176 NP fragments appear in >1000 clinical compounds published since 2008, yet these make up on average 63% of the clinical compound’s core scaffolds. There is untapped potential awaiting exploitation, by applying nature’s building blocks—“natural intelligence”—to drug design.

## Introduction

The historical application of molecules of natural origin as starting points for drug discovery^1^ has been largely replaced by today’s primary hit-seeking strategies of library screening (diversity-based, fragment-based, knowledge-based, and virtual) and exploitation of known compounds.^2,3^ Accordingly, the computed natural product (NP) probability score of approved oral drugs has fallen for drugs invented after 1990,^4^ when primary in vitro screening at cloned human targets began to be widely adopted. Despite this change, NPs, their derivatives, and synthetic compounds inspired by NPs make up 6, 28, and 33%, respectively, of all small molecule drugs approved from 1981 to 2019.^5^ This is consistent with the widespread appearance in approved drugs of NP partial structures and fragments.^6^ Opportunities for the re-emergence of NPs in drug discovery have been widely heralded.^1,4,7−11^

Pseudo-NPs (PNPs) provide a new approach to quantifying the appearance of NP structural elements.^12−14^ PNPs contain low-molecular-weight NP fragments, selected from a designed library,^15^ which are connected in defined ways not currently known to be achievable by biosynthetic pathways. The PNP concept has been validated by its appearance in the literature^16,17^ and by the design of several new classes of biologically active compounds.^18,19^ Thousands of PNPs are available from commercial sources, and a PNP screening library can be readily established.^17^

Some 90% of approved drugs have a Tanimoto similarity of >0.5 to their structurally closest human endogenous metabolite, and screening collections were found to be less “metabolite-like” than drugs.^20^ NP-likeness has been proposed^21^ to assist drug permeation via transporters, which evolved to facilitate entry of beneficial exogenous natural molecules, helping achieve selective tissue access and therapeutic efficacy. The aim of this work is to seek further support for the existence of “natural selection” in drug discovery. We employ a highly curated dataset^22^ from ChEMBL (version 32)^23^ to assess the impact of quantitative NP measures, including the presence of PNPs, in marketed drugs and phase 1–3 clinical compounds in comparison with a background of relevant, target-matched reference compounds.

## Results

### Methods

The general approach used is similar to our previous analysis of “drug-like” properties^24^ but with the following notable differences in the dataset assembly^22^: (1) a newer version of ChEMBL was used (version 32), with the focus on published literature information to observe time trends (excluding deposited data such as the patented kinase sets added to ChEMBL in 2013–16^22^); (2) clinical compounds in phases 1–3 as well as approved drugs are examined; (3) both clinical and background reference compound sets are carefully time-matched using first literature publication dates, with the emphasis here on recent practice (post-1990 and post-2008, see Results section); (4) the contemporary dataset is larger, with >1000 clinical compounds and drugs published since 2008 versus the 141 drugs first approved in 2010–2020 used previously.^24^

Approved drugs and phase 1–3 clinical compounds, with annotated biological targets understood to be responsible for their efficacy, here collectively called clinical compounds, were curated from ChEMBL version 32 as described.^22^ In addition, nonclinical compounds were curated, here called reference compounds, limited to those compounds with reported activity at the clinical compound’s targets.^22^ Reference compounds qualified for entry only if they had a recorded pChEMBL value for in vitro activity at one or more of the clinical compound’s targets; clinical compounds, already having published annotated targets, did not require pChEMBL values. Biological targets included mutated versions and considered the originating organism (95% were human targets); targets are defined in this article by unique “target name_mutant_organism” identifiers. Target classes were exhaustively identified^22^ and further consolidated here to 17 major groups. Kinases and G-protein coupled receptors (GPCRs, subdivided into aminergic, peptidic, and others based on their ligands) were the largest target classes, making up ∼25% each of the post-2008 dataset, followed by transferases, nuclear receptors, proteases, oxidoreductases, and 8 smaller target classes.

Because changes to drug properties over time are significant,^24,25^ we aimed to ensure that clinical and reference compounds were strictly compared in time-matched periods. A Journal publication date was necessary for all entries; for Reference compounds, this was extracted from ChEMBL directly. For clinical compounds, the dates of the first disclosure (normally the patent) were obtained from SciFinder (CAS SciFinder^n^, Chemical Compound Database | CAS) and used to analyze long-term trends. The first Journal publication dates of clinical compounds, where not in ChEMBL for post-1990 first disclosure compounds, were obtained from SciFinder. The median Journal publication date for post-1990 clinical compounds was five years after the first disclosure.

The full dataset after removal of molecules filtered by the PNP algorithm^17^ (predominantly molecular weight >1000 and presence of uncommon elements) contained 3173 unique clinical compounds and 388,027 unique reference compounds. There were 9644 clinical compound-target pairs and 596,341 reference compound-target pairs, covering 2285 targets. For the post-2008 Journal publication period, used for clinical versus reference compounds analysis (see Results section), there were 1212 unique clinical compounds and 229,569 unique reference compounds, comprising 2842 clinical compound-target pairs, 320,927 reference compound-target pairs, and 726 targets.

For analysis by individual target mean or median reference compound properties, we required the target to have ≥100 reference compounds.^22,24^ The post-2008 set contained 422 targets with ≥100 reference compounds, acted on by 1091 clinical compounds unique to each target class, comprising 2011 clinical compound-target pairs; 28 of the 1091 compounds (2.6%) are duplicated because they act at more than one target class. The range of reference compound counts acting at the clinical targets was 100–7484 (median of 408). Of the 1091 clinical compounds, 737 had one biological target, 132 had two targets, and 222 (122 acting at protein kinases) had three or more targets (range 3–17).

For quantitation of the NP character, three complementary measures were used:

1. **PNP_Status.** Compounds were assigned to one of four categories according to their NP fragment combination graphs.^16,17^ The NP library fragments used for this purpose are Murcko scaffolds^26^ (the core structures containing all rings without substituents except for double bonds, *n* = 1673) derived^16^ from a representative set of 2000 NP fragment clusters.^15^ Because of their ubiquitous appearances in NPs, the phenyl ring and glucose moieties were specifically excluded as fragments.^16^ The phenyl ring, however, does appear in some fragments, combined with other ring systems. The categories are:
  - **NP** (natural product). Naturally occurring compounds with defined structures and fragment combinations.
  - **NPL** (NP-like). Fragment connections appear as found in NPs, but the structures are different from NPs, e.g., NP derivatives, or compounds with additional NP fragments.
  - **PNP** (pseudonatural product). Two or more NP fragments linked by 0–3 atoms in defined ways not found biogenetically. Where NP fragment combinations in a molecule had both NP and PNP motifs, they were assigned to the PNP category.
  - **NonPNP**. All others.
2. **Frag_coverage_Murcko**. This measure has no dependency on the connectivity between fragments and is equal to the number of heavy (non-H) atoms (HA) present in the NP fragments divided by the total number of HA that are present in the Murcko scaffold of each molecule.
3. **NP-likeness**. A Bayesian measure of similarity to the structural space covered by natural products, calculated by the method of Ertl.^27^

PNP_Status is a compound categorization, whereas Frag_coverage_Murcko and NP-likeness have quantitative values for all compounds. Some illustrative examples of marketed drugs that are classified as PNP, NonPNP, and NPL, and the fragments they contain, are shown in Table 1.

**Table 1:**
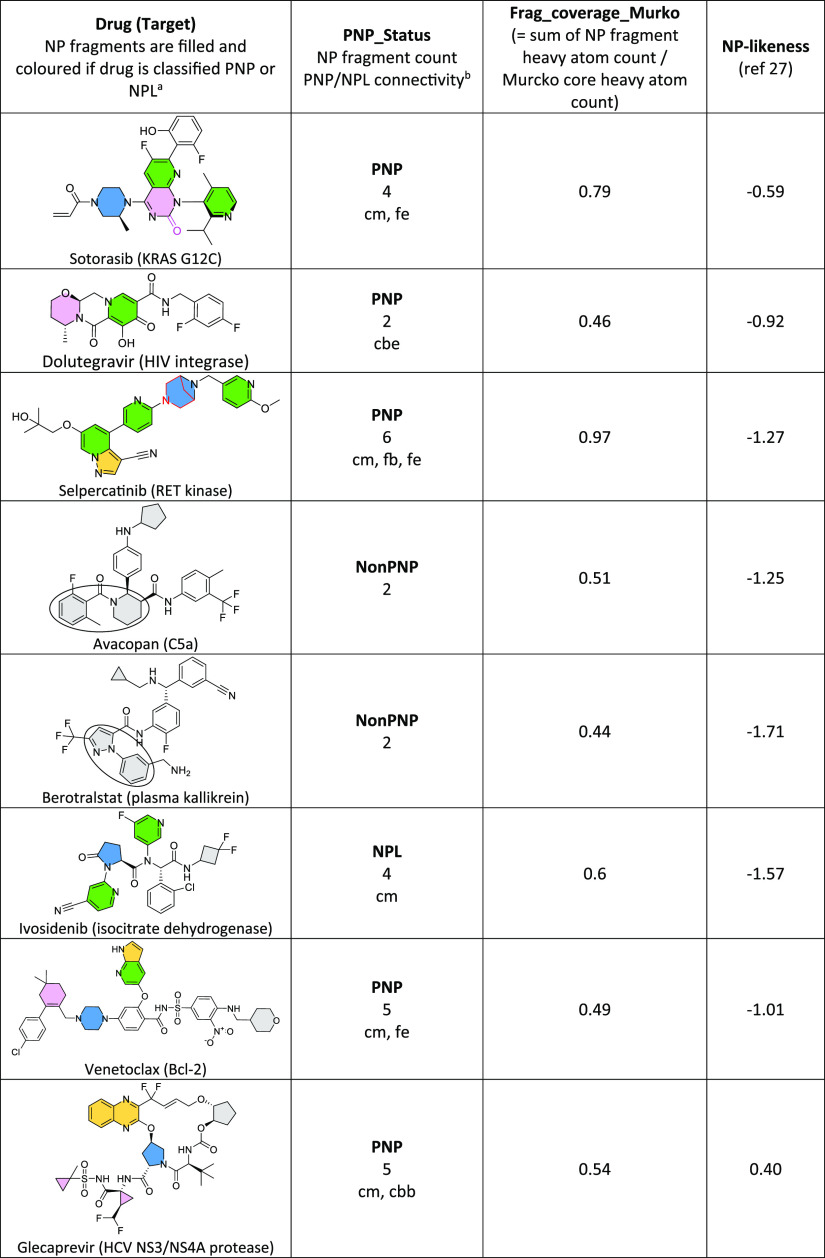
Examples of Marketed Drugs Illustrating the NP Metrics Used

a NP fragments are filled and those with >1 ring system are circled. Molecules with color-filled NP fragments are classified as pseudonatural products (PNP) or NP-like (NPL); see Methods section for details.

b In PNPs, NP fragments are connected in nonbiogenetically accessible ways (see ref (16) for complete definitions), using ≤3 atoms; in NPLs, NP fragments are connected biogenetically. The most common PNP connectivities seen in the post-2008 clinical compounds in this study are exemplified in the compounds shown: linear single bonds (termed connection monopodal, cm, 73.9%), ring fusion (termed fused edge, fe, 14.1%), bridged ring (termed fused bridge fb, 4.7%), and two bipodal options with one connecting ring (cbe, 2.2%; cbb, 1.4%). NonPNPs can contain NP fragments, connected by > 3 atoms.

In addition, the specific NP fragments, fragment combination pairs, and PNP fragment combinations that are found in clinical and reference compounds were identified in the post-2008 set, in full and by major target classes. Their relative clinical versus reference abundances were estimated using odds ratios (MedCalc’s Odds ratio calculator). Compound physicochemical properties were added to the dataset,^22^ taken from ChEMBL and RDKit.^28^

Calculations were performed using Microsoft Excel (https://www.microsoft.com/excel*)* and DataWarrior (www.openmolecules.org). Statistical significance (*p* values) was obtained from *t* tests assuming unequal variance for unpaired data or from Wilcoxon signed rank tests for paired data.

### Source Data Sets: Caveats and Limitations

#### NP Fragment Library

This was generated for use in fragment-based drug discovery (FBDD), with a focus on practical application and commercial fragment availability.^15^ Although not originally intended to be used for statistical analysis of NP characteristics, it is used as such here because it forms the basis of the PNP classification algorithm.^16,17^ The library was assembled starting from 183,769 NPs containing at least one ring, which contained 110,485 fragments.^15^ Using pharmacophore, physical property, and chemical alert filtering, this was reduced to 2000 representative clusters and further refined^16^ to a set of Murcko scaffold fragments. The NP library is composed of 1673 fragments (molecular weight 42–294) having either single ring systems (1421 fragments) or >1 ring system (252 fragments). Because of its targeted makeup, small size, and property distribution, the fragment NP library lacks NP acyclic substituents,^29^ and there is no directly comparable “non-NP” fragment set that could be used as a control set. Representation of all NP chemical ring systems is an impractical proposition. For example, other similar studies found 134,102 fragments (MW 100–300) including acyclic moieties in a set of 210,213 NPs,^6^ and 38,662 unique ring systems were found in 269,226 NPs, of which 23,299 (60%) were singletons.^30^ High diversity is apparent in studies of scaffold occurrence: singleton scaffolds dominate the medicinal chemistry literature^31^ and only 763 of 103,772 scaffolds (0.7%) in the ChEMBL 20 database were found in >10 compounds.^32^ Not surprisingly, high proportions of singleton NP fragments and singleton NP fragment combinations are also seen in the clinical and reference compound sets (see Results section). Of the 30 most abundant NP ring systems reported,^30^ 21 passed the fragment filtering process applied^15,16^ and are present in the NP fragment library.

#### ChEMBL Data

The freely available ChEMBL database abstracts comprehensive structure–activity data from the medicinal chemical literature and has become an established mainstay for chemoinformatic studies.^23^ As discussed earlier, here, we use only those reference compounds that are reported in scientific journals.^22^ A major caveat^22,24^ is that compounds revealed in publications are generally selected to illustrate how various problems were addressed and solved and, therefore, carry a risk of being unknowingly biased by author selection. A published medicinal chemistry case history is unlikely to be representative of all that was done, as in practice it discloses only a small proportion of all molecules synthesized in a project. Additionally, in describing structure–activity relationships, there may be a general tendency to emphasize more of the “better” compounds (more potent, good pharmacokinetics) than the “poorer” (less potent or metabolically unstable) compounds. While patented compounds are more likely to be representative of what was done, they often lack quantitative potency data, which we required for entry into the Reference compound set. Restricting the analysis to higher potency Reference compounds by introducing a pChEMBL cutoff could in principle improve quality. This was not done for two reasons: (1) it is not possible to compare relative potency values across very widely differing assay formats^33^ and (2) it would reduce the numbers of clinical compounds as well as clinical targets with ≥ 100 Reference compounds. Finally, the annotation of specific biological targets to clinical compounds is based on current knowledge and could change in the future.

#### Target Class NP Statistics

While trends in NP properties are clearly apparent (see Results section), statistically significant differences (*p* < 0.05) are often absent for individual target classes with lower numbers of clinical compounds and/or targets.

### Impact of Time on Clinical and Reference Compound NP Properties

**Figure 1:**
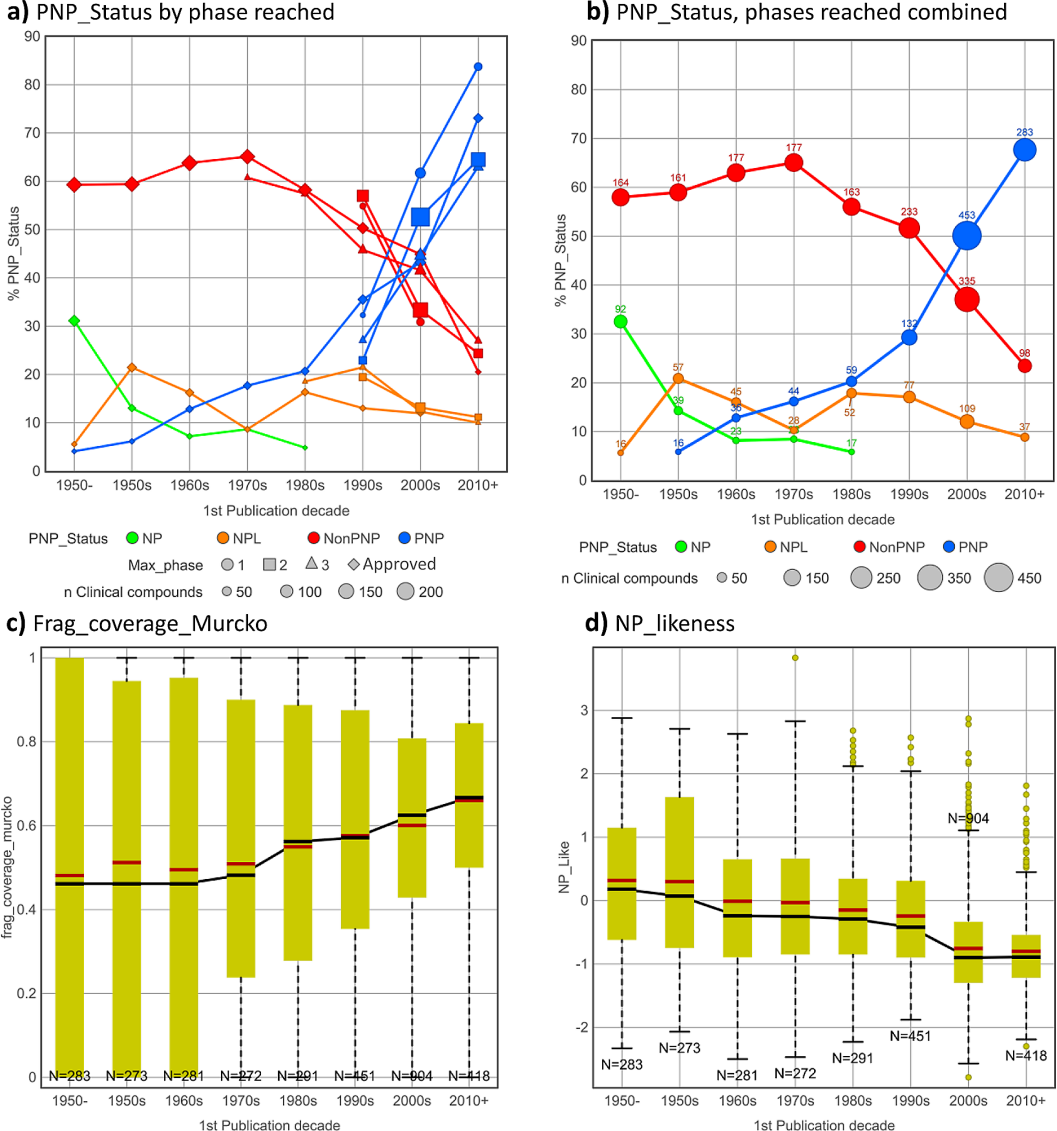
NP properties of clinical compounds by a decade of first publication (usually a patent). (a) % clinical compounds by phase reached in each PNP_Status category. (b) Same data as (a) with clinical phases combined. (c) Frag_coverage_Murcko. Significant increases (*p* < 0.05, unpaired *t* test) occur every second decade from the 1970s. (d) NP-likeness. Significant decreases (*p* < 0.05, unpaired *t* test) occur in the periods 1960s–1990s and 2000s onward.

**Figure 2:**
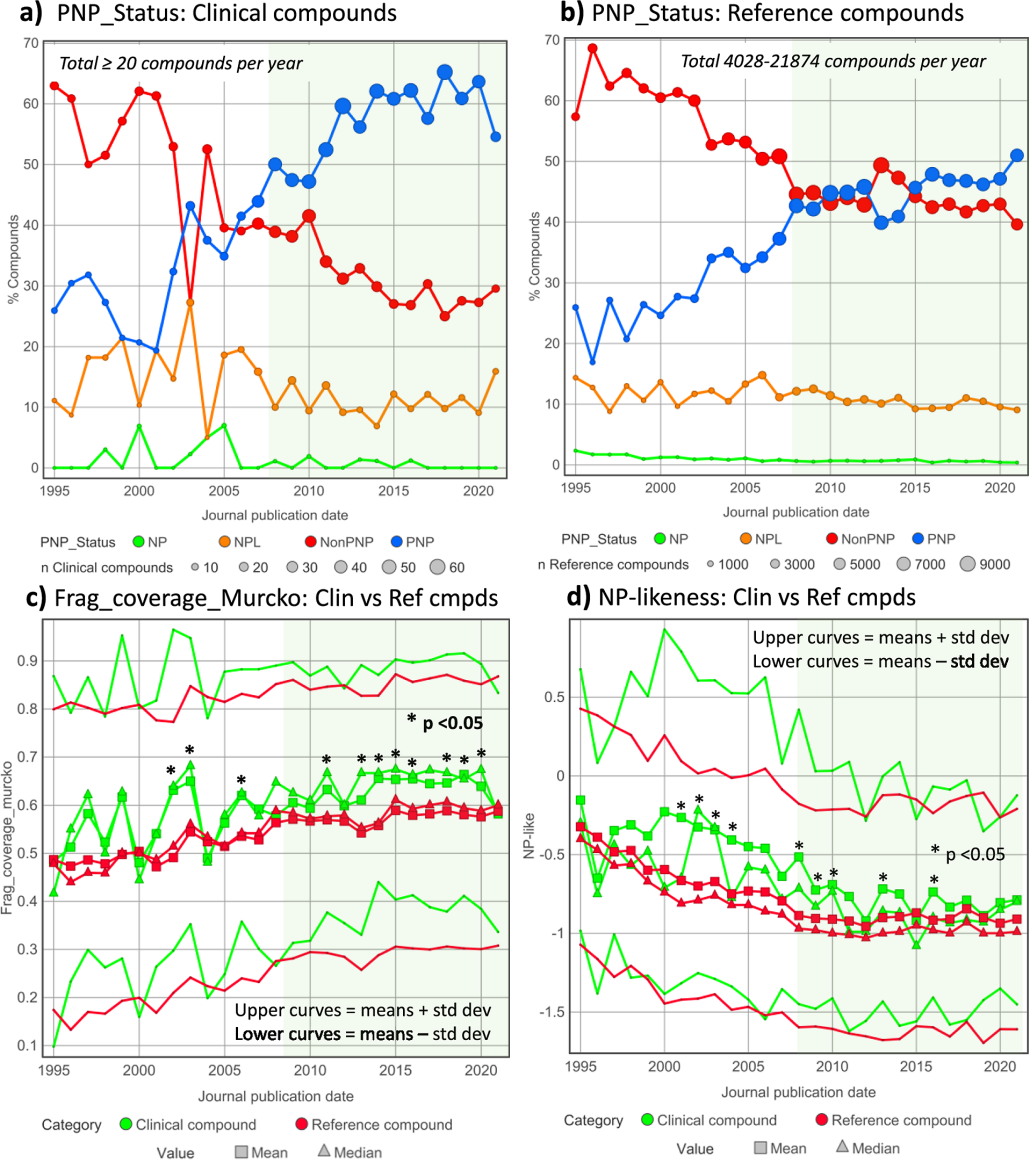
Clinical and reference compound NP properties by first Journal publication date since 1990. Clinical and reference compounds all act at the same targets. Clinical compounds all have first publication (usually patent) dates 1990 or later (the years 1990–1994 have <20 clinical compounds and are not shown). (a) % Clinical compounds in each PNP_Status category. (b) % Reference compounds in each PNP_Status category. (c) Frag_coverage_Murcko. (d) NP-likeness. *p* Values are from *t* tests assuming unequal variance. Reference compound NP values do not alter post-2008 (shaded green), and this period was chosen for target class analysis.

The long-term progression of the fraction of clinical compounds by phase reached in each of the PNP_Status categories (Figure 1a) shows near-identical time trends in all clinical phases (1–3 and approved drugs), so clinical compounds were combined into a single group for further analysis (Figures 2b–d). The consistent increase in PNP fraction is striking, approximately doubling every 2–3 decades, reaching 67% of all clinical compounds in the 2010s (Figures 2a,b). Increases in Frag_coverage_Murcko values have occurred every second decade, with their distribution narrowing over time: after 2010 the average clinical compound has a value of 0.66, with first and third quartile values of 0.50 and 0.84, respectively (Figure 1c). The fraction of NP compounds is falling over time (Figure 1b), which is consistent with the trend in NP-likeness, which falls significantly from historical levels in the 1960s–1990s, and further again from 2000 onward (Figure 1d).^4^ Interestingly, NPL compounds consistently appear in all time periods at ∼10 to 20% of the total (Figure 1b). NonPNPs were the majority class until the 1990s; because PNPs and NonPNPs are the dominant classes overall, their fractional occurrence over time is inversely related. It is clear from Figure 1 that while application of NP structures per se has declined markedly, the “NP signal” is nevertheless present, expressed by the increasing application of NP-derived fragments, resulting in the current dominance of PNP clinical compounds.

**Figure 3:**
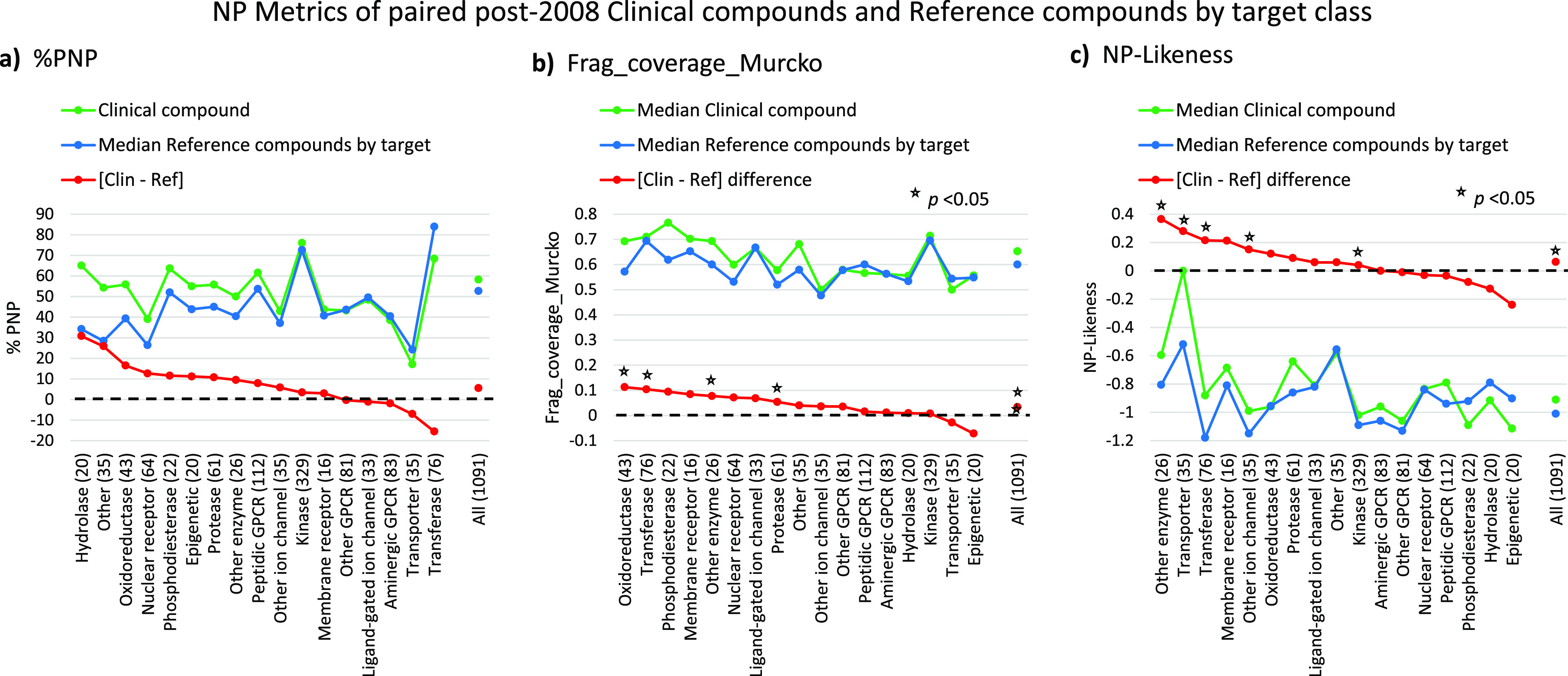
Post-2008 paired clinical versus target reference compound analysis of NP metrics by target class. Clinical compounds in each target class are unique, and paired with their corresponding reference compounds by target(s). Target values are median values for all targets having ≥100 reference compounds. Target classes on the *x*-axis are shown with the numbers of clinical compound-target pairs. (a) %PNP; this is a categorical measure, and the clinical-reference differences (in red) are the arithmetic differences. (b) Frag_coverage_Murcko. (c) NP-likeness. For (c, d), the clinical-reference differences are the medians of the differences for each clinical compound. The black dotted line is where clinical and reference values are equal. The rank orders of target class [clinical-reference] difference values differ according to the NP metric used. The correlation *r*^2^ values (*n* = 17) are: 0.003 for % PNP vs Frag_coverage_Murcko; 0.181 for % PNP vs NP-likeness; and 0.187 for Frag_coverage_Murcko vs NP-like. *p* Values were determined using the Wilcoxon signed rank test.

The period from 1990 onward was selected for comparing clinical with reference compounds by NP properties because of the sharp increase in numbers of clinical PNPs (Figure 1b), and the availability of several thousand comparative ChEMBL reference compounds each year. Figure 3a,b shows the progression of the PNP_Status categories of the post-1990 clinical compounds present in Figure 1b, and target-matched ChEMBL reference compounds, both by the first Journal publication date. While PNP fraction increased in both clinical (Figure 2a) and reference (Figure 2b) compounds until ∼2008, reference compounds show a markedly smaller increase than clinical compounds. Comparing Figure 3a,b shows a clear “enrichment” of PNPs in clinical compounds versus reference compounds in the post-2008 period. It is also apparent from Figure 3a,b that the fractions of compounds in the NP and NPL categories are very similar in clinical and reference compounds and do not change over time.

Consistent with the PNP time trend, Frag_coverage_Murcko (Figure 2c) values are increasing over time, with clinical compounds having significantly higher values than reference compounds in eight years after 2010. NP-likeness is decreasing over time, as expected, in both clinical compounds compared to reference compounds (Figure 2d). However, higher NP-likeness values for clinical compounds versus reference compounds are seen in nine years after 2000, although the differences are less pronounced in the post-2010 period. Comparing all clinical and reference compounds since 2008 confirms higher values in NP properties for clinical compounds (Table 2). By odds ratio analysis of the full dataset, a post-2008 clinical compound is 54% more likely to be a PNP than a reference compound (Table 2).

**Table 2:** NP Property Values for Clinical and Reference Compounds for the Full Post-2008 Set

(a) % PNP and clinical vs reference odds ratio					
category	clinical count (%)	reference count (%)	odds ratio	95% CI	*p*
PNP	666 (57.3%)	101925 (46.6%)	1.54	1.37–1.73	<0.0001
other	497 (42.7%)	116848 (53.4%)			

(b) NP properties: clinical vs reference compounds					
property	category	mean	median	std. dev.	*p*
Frag_coverage_Murcko	clinical	0.62	0.64	0.26	<0.0001
	reference	0.58	0.59	0.28	
NP-likeness	clinical	–0.77	–0.9	0.74	<0.0001
	reference	–0.92	–1	0.71	

Collectively, Figure 2 indicates that clinical compounds possess greater “NP character,” based on the three measures used, than the reference compounds. Notably, for each of the NP metrics, there is relatively little change in their reference compound annual values from 2008 onward (shaded green in Figure 2). For this reason, the post-2008 period was selected for further analysis by target class.

### Post-2008 Target Class NP Properties

The results in Figures 1, 2 and Table 2 take no account of the biological targets followed, which are known to influence the physical properties of their ligands.^24,34^ The role of each target class (*n* = 17) on the NP profiles of post-2008 clinical versus reference compounds was examined in three ways:

1. Comparisons of all clinical (*n* = 1212) versus all reference (*n* = 229,569) compounds. This takes no account of the widely differing numbers of reference compounds annotated to each target. As an example, in the kinase target class, this approach compares 330 clinical compounds with 50,370 reference compounds.
2. Unpaired comparisons of all clinical compounds (*n* = 1091) versus the median values for their targets that possess ≥100 reference compounds (*n* = 422). This treats reference compounds on an equal footing by target but does not completely account for clinical compounds that have multiple targets. In the kinase target class example, 329 clinical compounds are compared with the median values for 122 single kinase targets having ≥100 reference compounds.^22,24^
3. Paired comparisons of all clinical compounds versus the corresponding median values for their targets that possess ≥100 reference compounds (*n* = 1091).^24^ While this results in certain target values being duplicated (especially kinases), it reflects the reality that some targets have been pursued more than others. For those clinical compounds with >1 target (354 of 1091, 32.4%), medians of the target median NP property values were used. In the kinase target class example, 329 clinical compounds are compared with the 329 median values for their annotated targets having ≥100 reference compounds.

It is apparent from the results of the paired analysis (i.e., 3 above), as shown in Figure 3, that target class influences all the NP properties and that, for the majority, clinical compounds have higher NP property values than the corresponding reference compounds. In addition, the rank orders of target class [clinical-reference] differences are different for each NP metric in Figure 3, suggesting that the three measures are complementary estimates of NP character.

The corresponding analyses by all compounds and by unpaired targets (i.e., (1) and (2) above) are qualitatively very similar to Figure 3 (Figure S1). The collected % differences in the NP measures between clinical and reference compounds found by the three approaches (Table 3) show that increased NP properties in clinical compounds versus reference compounds consistently appear more frequently by target class than the opposite possibility. The three NP measures show differing results by target classes; for example, epigenetic and transporter display opposite trends on % PNP and NP-likeness. In the case of clinical transporter compounds, the NP-likeness values are increased by a group of 12 sodium/glucose cotransporter 2 (SGCL2) inhibitors (e.g., empagliflozin), which all possess a glucose part-structure (excluded as an NP fragment^16^). Across target classes, there is clear variability in PNP content with protein kinases and transferases being the most PNP-rich. Most kinase inhibitors bind competitively to the adenosine triphosphate (ATP) site, mimicking the adenosine heterocycle’s hydrogen bond donor and/or acceptor interactions with the “hinge” kinase domain. With transferases, the target diversity is rather limited as it is dominated by compounds acting at the four isoforms of phosphoinositol-3-kinase (PI3Kα, β, γ, and δ), which explains the different % PNP values in the paired and unpaired targets (Table 3).

**Table 3:**
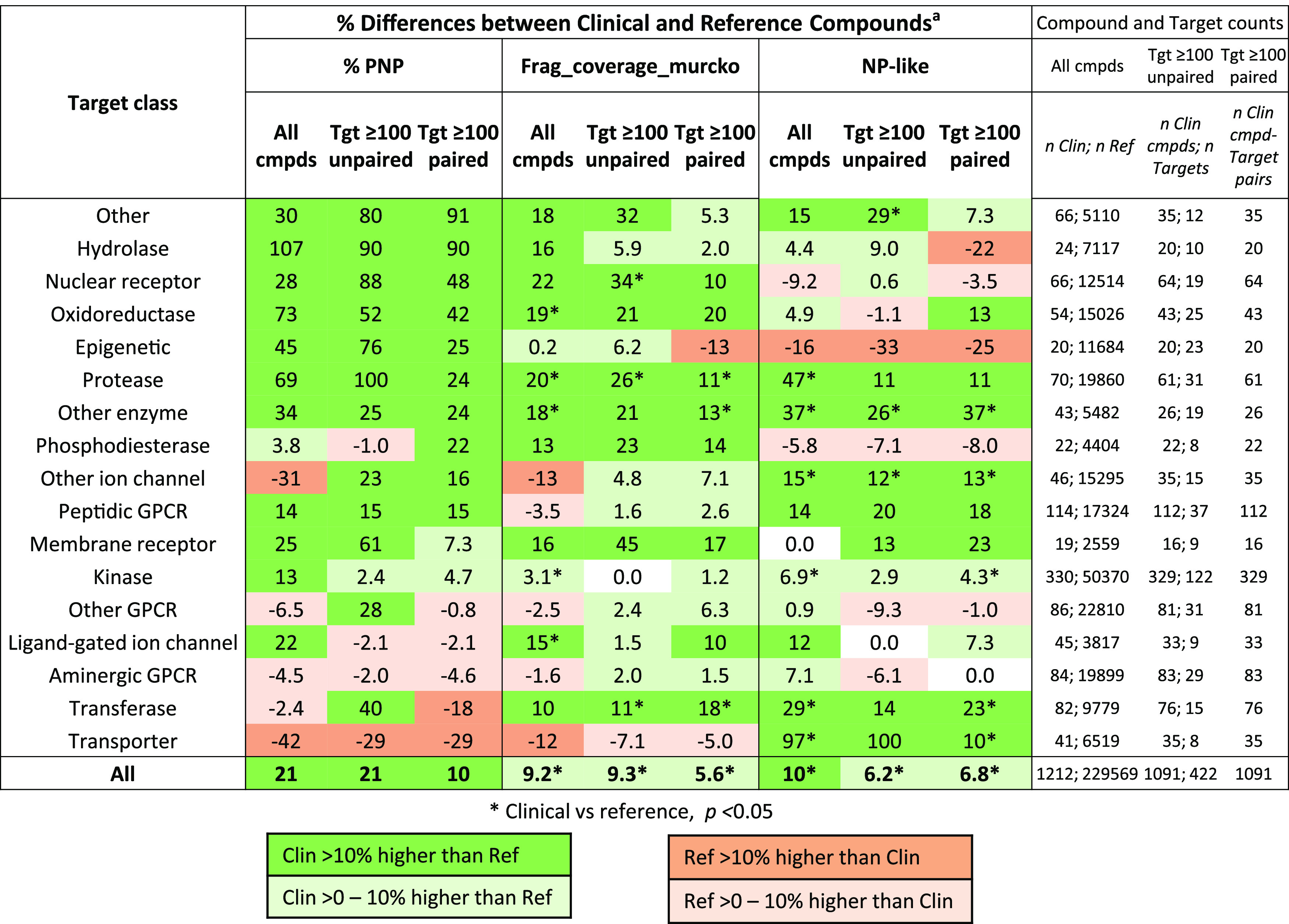
Summary of Clinical vs Reference Compound Normalized NP Property Trends by Target Class

a The color-coded % differences between clinical and reference compound median NP properties are shown and found using (1) all compounds, and clinical compounds either (2) unpaired or (3) paired to targets containing ≥100 reference compounds (see Results section). The % values shown are equal to [(clinical ÷ reference) – 1)] × 100 for % PNP and Frag_coverage_Murcko, and because all values are negative, [−(clinical ÷ reference) + 1)] × 100 for NP-likeness. The table is ranked by the paired % PNP score. The color coding provides a qualitative guide to clinical or reference compound preference. * *p* < 0.05 (Wilcoxon signed rank test).

Unsurprisingly, G-protein-coupled receptors (GPCRs) and protein kinases dominate the dataset, making up ∼ 25% of each of the total numbers of clinical and reference compounds. GPCRs are divided into three subclasses, aminergic, peptidic, and other, based on their endogenous agonists. It is clear from Table 3 that the kinases show only relatively minor NP enrichment in Clinical compounds, and the aminergic GPCRs virtually none. Clinical compounds acting at peptidic GPCRs in contrast have qualitatively higher % PNP and NP-likeness than their reference compounds. Overall, there is no obvious link between the clinical NP preferences of target classes and the number of clinical compounds contained therein.

### Post-2008 NP Properties versus Physicochemical Properties

**Figure 4:**
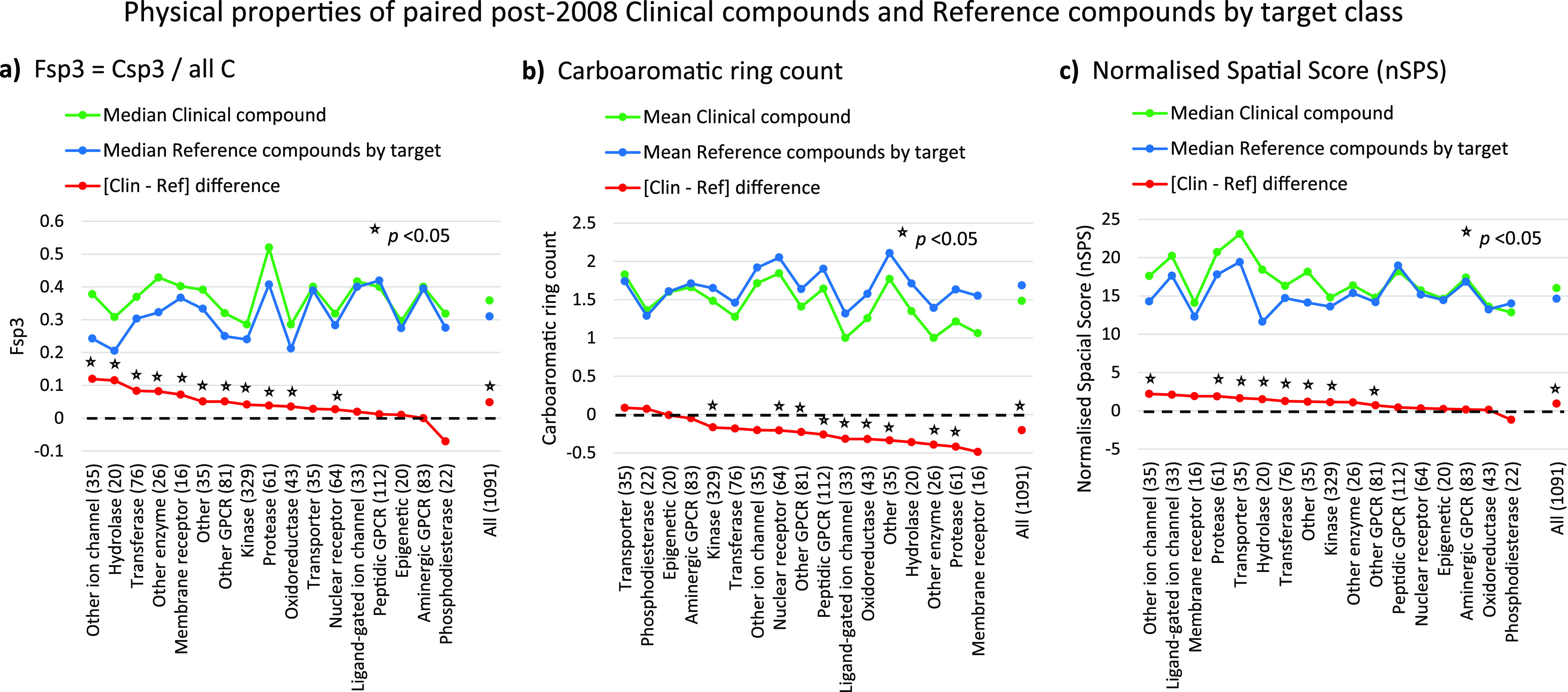
Benchmarked physical properties using the paired clinical compound and reference compounds by target set, for comparison with the corresponding NP properties shown in Figure 3. *p* Values are from Wilcoxon signed rank tests.

The NP results by target class were benchmarked by comparison to corresponding physical property trends. The results recapitulate the main conclusions of our earlier study using fewer clinical compounds.^24^ The most statistically significant and consistent differences between clinical and reference compounds by target class were carboaromatic ring count (lower in clinical compounds), the fraction of carbon sp3 atoms (Fsp3) and the number of stereocenters (both increased in clinical compounds). The Fsp3 and carboaromatic ring results are shown in Figure 4, together with the normalized spatial score (nSPS),^35^ a measure of complexity that takes stereocenters and other topological features into account. Comparing Figures 3 and 4 indicates that the NP measures by target class are qualitatively similar to these physical properties as indicators of differences between clinical versus reference compounds.

Fsp3 and stereocenter count are known to be greater in NP and naturally inspired drugs, and aromatic ring count is lower, versus synthetic drugs.^36,37^ However, the [clinical-reference] differences for the NP properties of all clinical compounds (*n* = 1091) are not highly correlated with the corresponding values for these three properties, or for a range of other physicochemical properties (Table S1). Similarly weak correlations are also seen using the target class data (*n* = 17, not shown). This is further evidence that the three NP metrics can be considered as independent measures of clinical compound quality.

Consistent with their defined makeup, PNPs contain on average two more NP fragments than NonPNPs (illustrated for post-2008 clinical compounds in Table 4; see Table S2 for a full summary of the physical properties examined). Accordingly, PNPs and NonPNPs have markedly different ring counts, both aromatic and aliphatic. The number of heteroaromatic rings and aromatic nitrogen atoms is more than doubled in PNPs versus NonPNPs, while there are fewer carboaromatic rings in PNPs (Table 4). Aliphatic ring counts are also significantly increased in PNPs versus NonPNPs. Additionally, PNPs have one additional H-bond acceptor and one fewer rotatable bond versus NonPNPs. Notably, Fsp3 and numbers of stereocenters, which differ between clinical and reference compounds, are not different between PNPs and NonPNPs (Table S2). This observation reinforces the independence of PNP fraction from these physical properties in distinguishing clinical from reference compounds.

**Table 4:** Physical Properties of Post-2008 Clinical PNPs and NonPNPs

property^a^	PNP (*n* = 666)	NonPNP (*n* = 368)	mean PNP versus mean NonPNP	
	mean, median (std. dev.)		difference	% relative difference^b^
NP fragments	3.17, 3 (1.01)	1.22, 1, (0.80)	2.05	159%
Frag_coverage_Murcko	0.74, 0.75 (0.18)	0.38, 0.38 (0.24)	0.36	93%
NP-like	–0.89, −0.97 (0.65)	–0.70, −0.85 (0.72)	–0.19	–27%
carboaromatic rings	1.35, 1 (0.89)	1.71, 2 (0.86)	–0.36	–21%
heteroaromatic rings	1.86, 2 (1.01)	0.73, 1 (0.72)	1.13	155%
aromatic N atoms	2.80, 3 (1.72)	1.13, 1 (1.32)	1.67	149%
carboaliphatic rings	0.47, 0 (0.85)	0.24, 0 (0.77)	0.23	97%
heteroaliphatic rings	1.06, 1 (1.02)	0.66, 1 (0.77)	0.40	60%
rotatable bonds	5.80, 5 (2.74)	6.93, 6 (3.79)	–1.13	–16%
H-bond acceptors	6.79, 7 (2.17)	5.36, 5 (2.19)	1.46	27%
PSA	98.7, 95.3 (34.6)	87.8, 88.4 (35.6)	10.9	12%
mol. wt, ALogP, cx_LogD, H-bond donors, stereocenters, Fsp3, pChEMBL, LE, LLE, QED				–10 to 10%

a Values shown are for properties that differ between PNPs and NonPNPs by >10%.

b % Relative difference = [(PNP/NonPNP) – 1] × 100 and for NP-like, [−(PNP/NonPNP) + 1] × 100. *p* < 0.0001 in each case, by *t* tests assuming unequal variance.

All clinical compounds (*n* = 3173), contain 937 ring systems (using ring systems from DataWarrior’s scaffold calculator^38^). New ring systems added per decade account for approximately 30% of all ring systems present, in agreement with a recent analysis.^39^ However, clinical PNPs add more new ring systems than the clinical NonPNPs, and the proportion of new ring systems is increasing over time in PNPs, but not NonPNPs (Figure S2). PNP ring system novelty versus NonPNPs is consistent with their higher aliphatic and heteroaromatic ring counts (Table 4).

### NP Fragments and Fragment Pair Combinations Present in Clinical and Reference Compounds

Counts of all NP fragments, fragment pair combinations, and PNP fragment pair combinations occurring in the post-2008 clinical and reference compounds are summarized in Table 5. The fragment pair combinations can have more than one of each fragment types present. Of the 1673 individual fragments in the NP library, it is notable that only 176 (10.6%) are used in clinical compounds, and a further 296 (17.7%) are unique to reference compounds (Table 5). However, the 176 clinical fragments comprise 97.8% of the total fragment coverage seen in the reference compounds. For the much higher numbers of fragment pair combinations, there is lower coverage by clinical compounds (e.g., 72.8% for all fragment pairs and 63.1% for all fragment pair PNP combinations, Table 5) and reference-only fragment pairs outnumber clinical fragment pairs by up to 10-fold across major target classes (e.g., GPCRs, Table 5). Reference singleton counts are dominant in both classes of fragment pairs.

**Table 5:** Total Fragment Counts in Clinical and Reference Compounds for the Post-2008 Set

group	major target class^a^	*n* clinical (% occurrence in reference)	*n* clinical only	*n* clinical singletons	*n* reference only (% occurrence	*n* reference singletons
fragments	all	176 (97.8%)	2	53	296 (2.1%)	61
	GPCR	86 (93.5%)	1	26	197 (6.5%)	26
	kinase	85 (96.8%)	1	26	178 (3.2%)	42
	other	146 (95.4%)	1	46	277 (4.6%)	55
fragment pair combinations	all	1074 (72.8%)	53	571	6372 (27.2%)	1855
	GPCR	318 (49.2%)	12	214	3542 (50.8%)	918
	kinase	487 (66.7%)	14	276	2796 (33.3%)	839
	other	669 (59.9%)	50	410	5093 (40.1%)	1566
fragment pair PNP combinations	all	842 (63.1%)	67	534	5748 (36.9%)	1745
	GPCR	223 (39.0%)	21	169	2583 (61.0%)	802
	kinase	363 (58.2%)	24	243	2447 (41.8%)	774
	other	462 (45.9%)	47	324	4111 (54.1%)	1365

a Clinical and reference compound counts: all, 1163 and 218773; GPCR, 284 and 59632; kinase, 330 and 50370; other, 574 and 115851.

**Figure 5:**
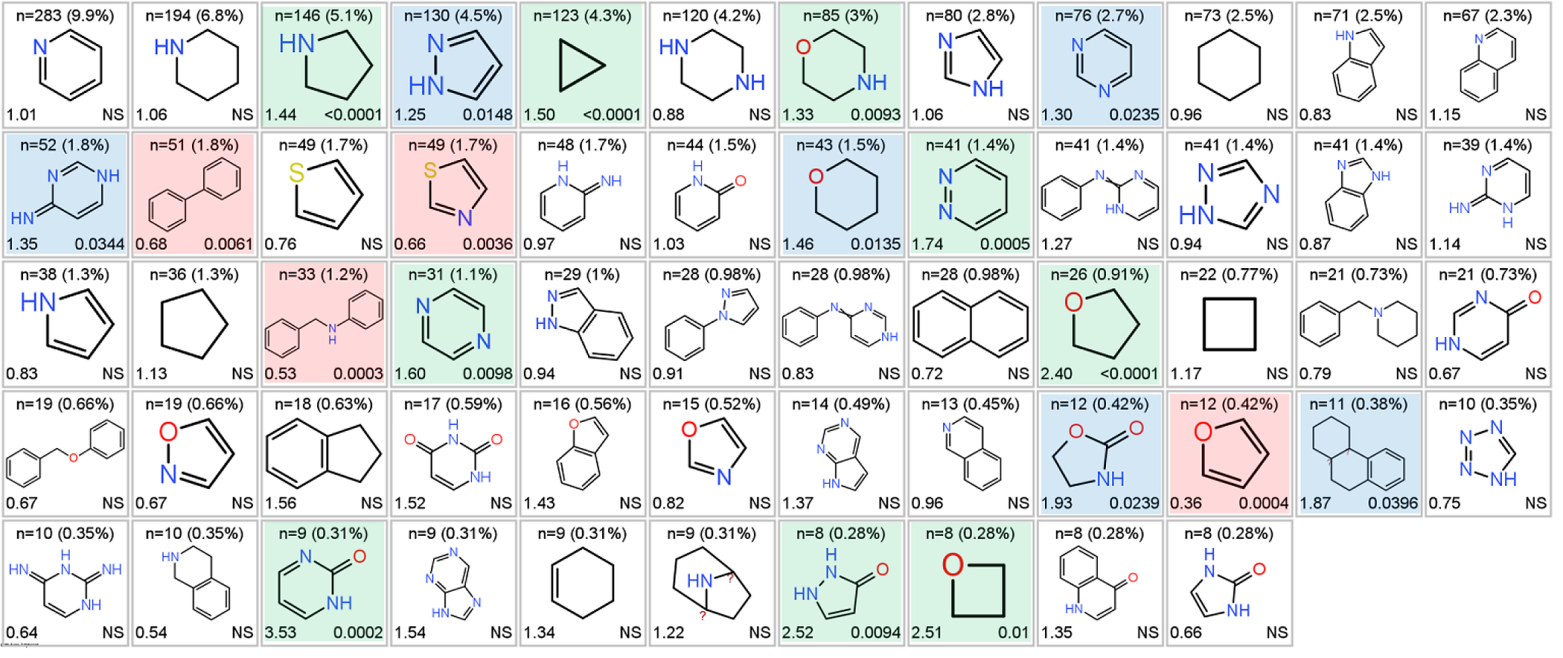
58 Most abundant post-2008 clinical NP fragments (≥8 occurrences, 90.5% of total clinical NP fragment count). Shown for each fragment are: count (% clinical fragments) (top); odds ratio vs reference compounds (lower left); *p* value (lower right; NS = not significant, *p* > 0.05). Clinical abundance increased (15, 26%): green, *p* < 0.01; blue, *p* = 0.01–0.05). Clinical abundance decreased (4, 7%): red (pink, *p* < 0.05). Canonical tautomers are shown, as generated by RDKit.

**Figure 6:**
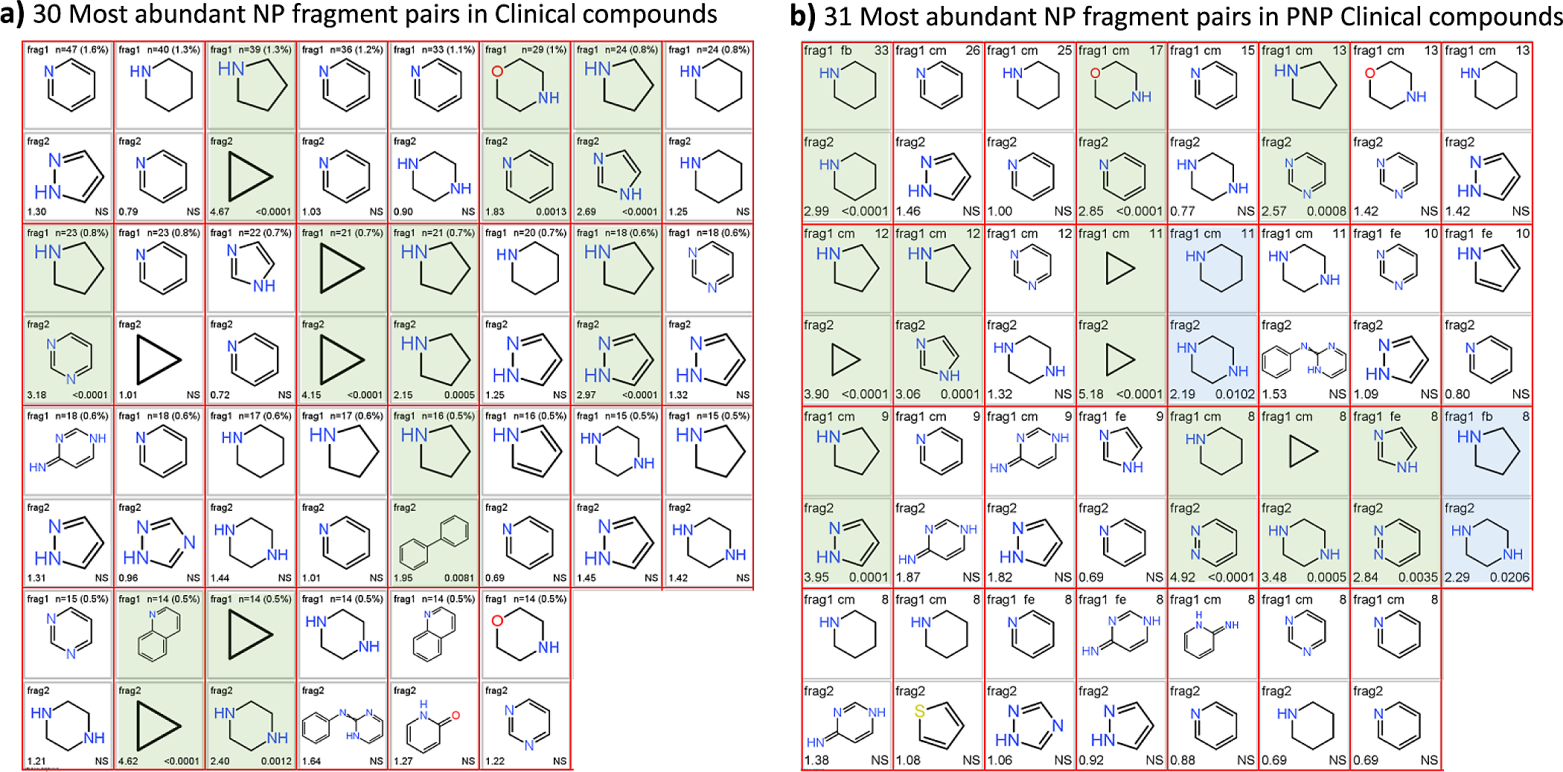
(a) 30 Most abundant fragment pair combinations all post-2008 clinical compounds (≥14 occurrences, 21.6% of all fragment pair occurrences). (b) 31 Most abundant fragment combinations in all PNP clinical compounds (≥8 occurrences, 22.0% of all PNP fragment pair occurrences). Shown are fragment pairs, count (%), odds ratio for clinical vs reference compounds, and *p* value. Connectivity definitions (see ref (16) between fragments in b) are: cm, connection monopodal (single bond connections); fb, bridge fused connections, 3–5 fused atoms; fe, fused edge connections, 2 fused atoms. Clinical abundance increased: green (*p* < 0.01), blue (*p* < 0.01–0.05). Canonical tautomers are shown, as generated by RDKit.

The occurrence of the most common fragments and fragment combinations seen in all post-2008 clinical compounds was compared with that seen in the reference compounds. The 58 most common fragments, occurring ≥8 times in clinical compounds (Figure 5), account for 90.5% of the total clinical fragment occurrence. Of the 58 fragments, 15 (26%) occur more frequently in clinical versus reference compounds (by odds ratio, *p* < 0.05), and 4 (7%) are increased in reference compounds (Figure 5). The 30 most common clinical fragment pair combinations (Figure 6a), occurring ≥14 times, account for 22% of the total clinical fragment occurrence, and 10 of these (33%) are significantly more abundant clinically. Similar clinical preferences are seen for the 31 most common PNP combinations, occurring ≥8 times and accounting for 22% of the total clinical occurrence (Figure 6b). It is apparent from Figures 5 and 6 that the pyrrolidine and cyclopropyl rings feature prominently, individually and in combination with other fragments, as having higher abundance in clinical than in reference compounds. The presence of NP fragments that are enhanced in reference compounds, namely biphenyl, thiazole, N-benzylaniline, and furan (Figure 5) indicates that not all NP fragments are necessarily ideal, indeed some may possess liabilities. However, biphenyl is enhanced in clinical compounds when in combination with pyrrolidine (5th entry, row 3, Figure 6).

### PNP Trends over Time in Selected Targets

**Figure 7:**
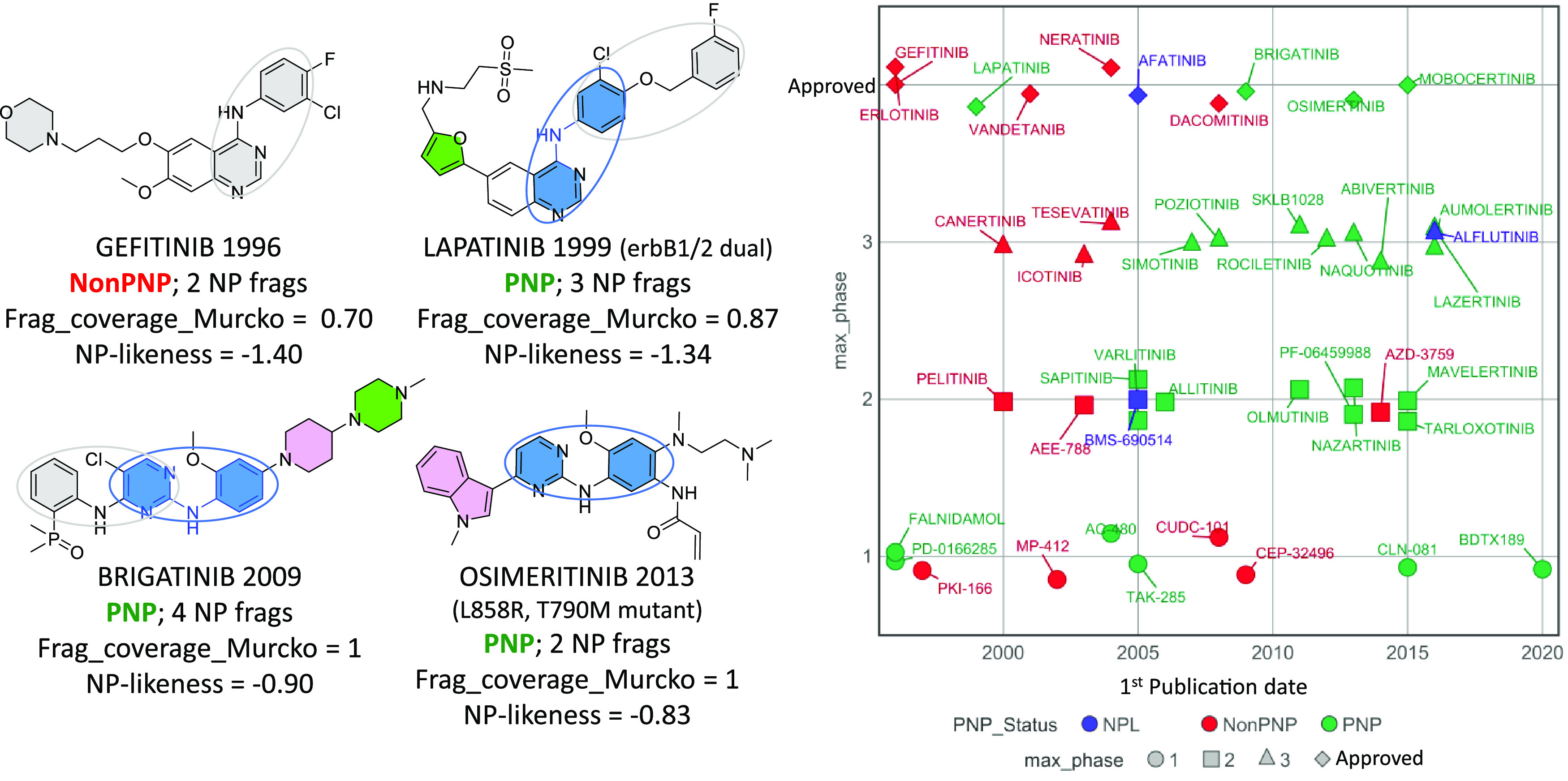
Development of EGFR-1 (erbB1) antagonists. NP fragments are filled and those with >1 ring system are circled. Color-filled NP fragments show molecules classified as PNPs. In all, 26 of 44 clinical compounds since 1995 are PNPs (59%) and 2899 of 5480 reference compounds are PNPs (53%); odds ratio = 1.29 (*p* > 0.05)). Since 2005, 22 of 29 clinical compounds are PNPs (76%) and 1499 of 3193 reference compounds are PNPs (47%); odds ratio = 3.65 (*p* = 0.0036).

The overall increase in PNP clinical compounds (Figure 1a,b) is evident in heavily pursued targets that have seen long-term production of clinical compounds. For the epidermal growth factor receptor-1 (EGFR-1) kinase (Figure 7), the invention of the 45 clinical compounds found in ChEMBL has spanned 30 years. The first drug to the market, gefitinib, is not a PNP but contains two NP fragments, one of which, 4-anilinopyrimidine, inspired further development of the class, leading to approved PNPs such as lapatinib, followed later by brigatinib and osimertinib, which both contain a 2-anilinopyrimidine NP fragment (Figure 7). PNPs have been the dominant class of EGFR clinical compounds since 2005, with their occurrence exceeding that seen in reference compounds (Figure 7). Vascular endothelial growth factor receptor 2 (VEGF-2) kinase, with 54 clinical candidates over 26 years, shows a similar increase in PNP clinical compounds over time (Figure S3).

**Figure 8:**
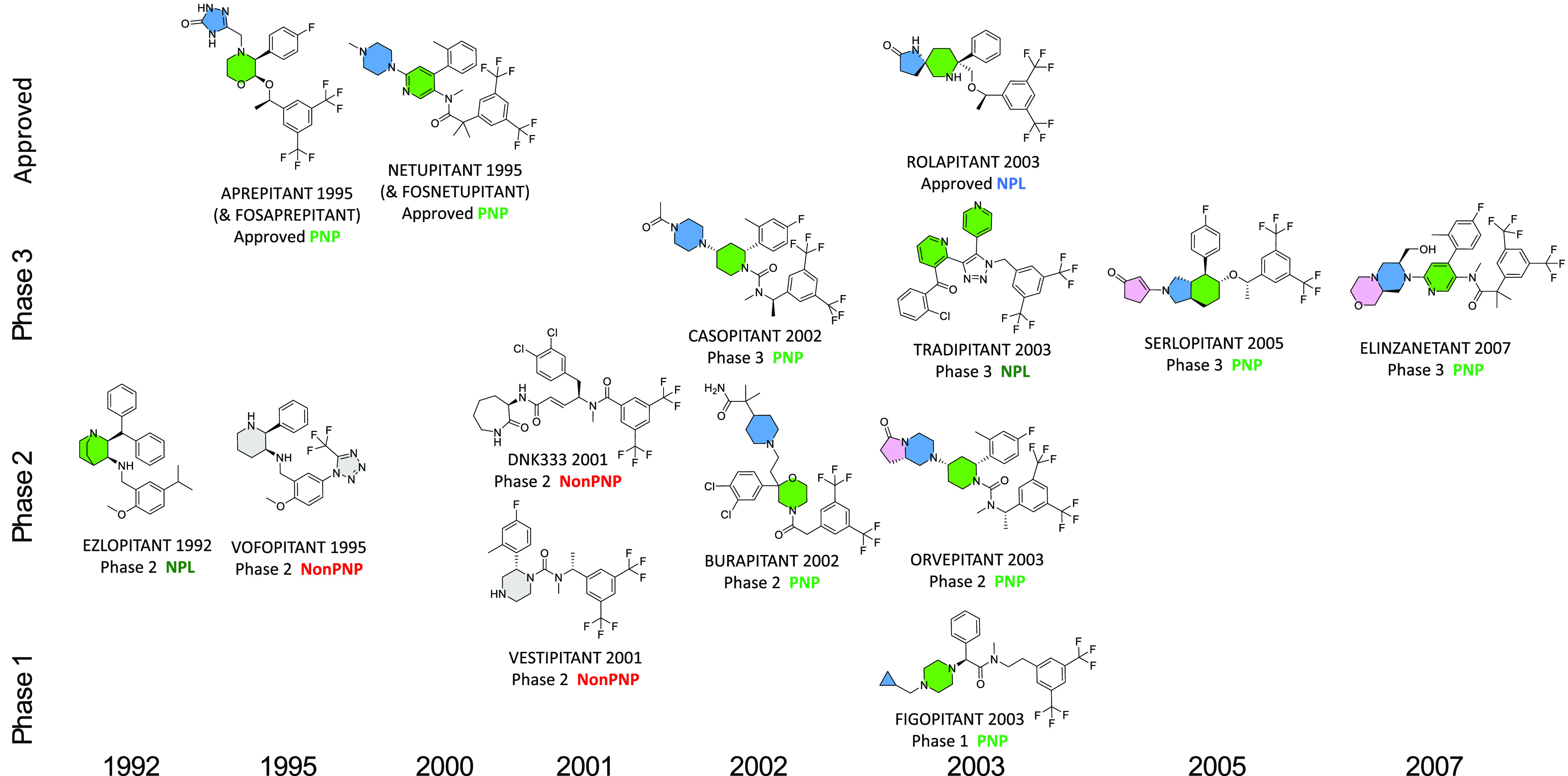
Development of the clinical NK-1 antagonists. NP fragments are filled. Color-filled NP fragments show molecules classified as PNPs or NPL; PNP occurrence in clinical compounds (10/16, 63%) exceeds that seen in reference compounds (470/2158, 22%), with an odds ratio of 5.99 (*p* = 0.0006).

Unsurprisingly, among targets having multiple clinical compounds, exploitation of the first molecules discovered is common practice, leading to subsequent molecules that often share comparable pharmacophoric features, a tendency that can also lead to promulgation of the same PNP_Status. An example is the neurokinin-1 (NK-1) receptor, a peptidic GPCR, which provided 16 clinical compounds in the ChEMBL database (with five marketed, including two prodrugs) disclosed between 1992 and 2007. All NK-1 antagonists that reached Phase 3, and all discovered after 2002, are classified as NPL or PNP (Figure 8). Following aprepitant in 1995, all subsequent clinical NK-1 antagonists employed a common 3,5-difluoromethylphenyl group and an additional phenyl ring. Aprepitant and netupitant bind to NK-1 in a similar mode,^40^ except for the pendant heterocycles that are part of their PNP motifs (triazolone and piperazine, respectively, Figure 8). However, both compounds are biologically comparable since they induce an identical intrahelical H-bonding network in the NK-1 structure, which might explain the advantageous insurmountable antagonism they exhibit.^40^ The NK-1 receptor stands out as a target where PNP occurrence in clinical compounds (10/16, 63%) exceeds that seen in reference compounds (470/2158, 22%), with an odds ratio of 5.99 (*p* = 0.0006).

**Figure 9:**
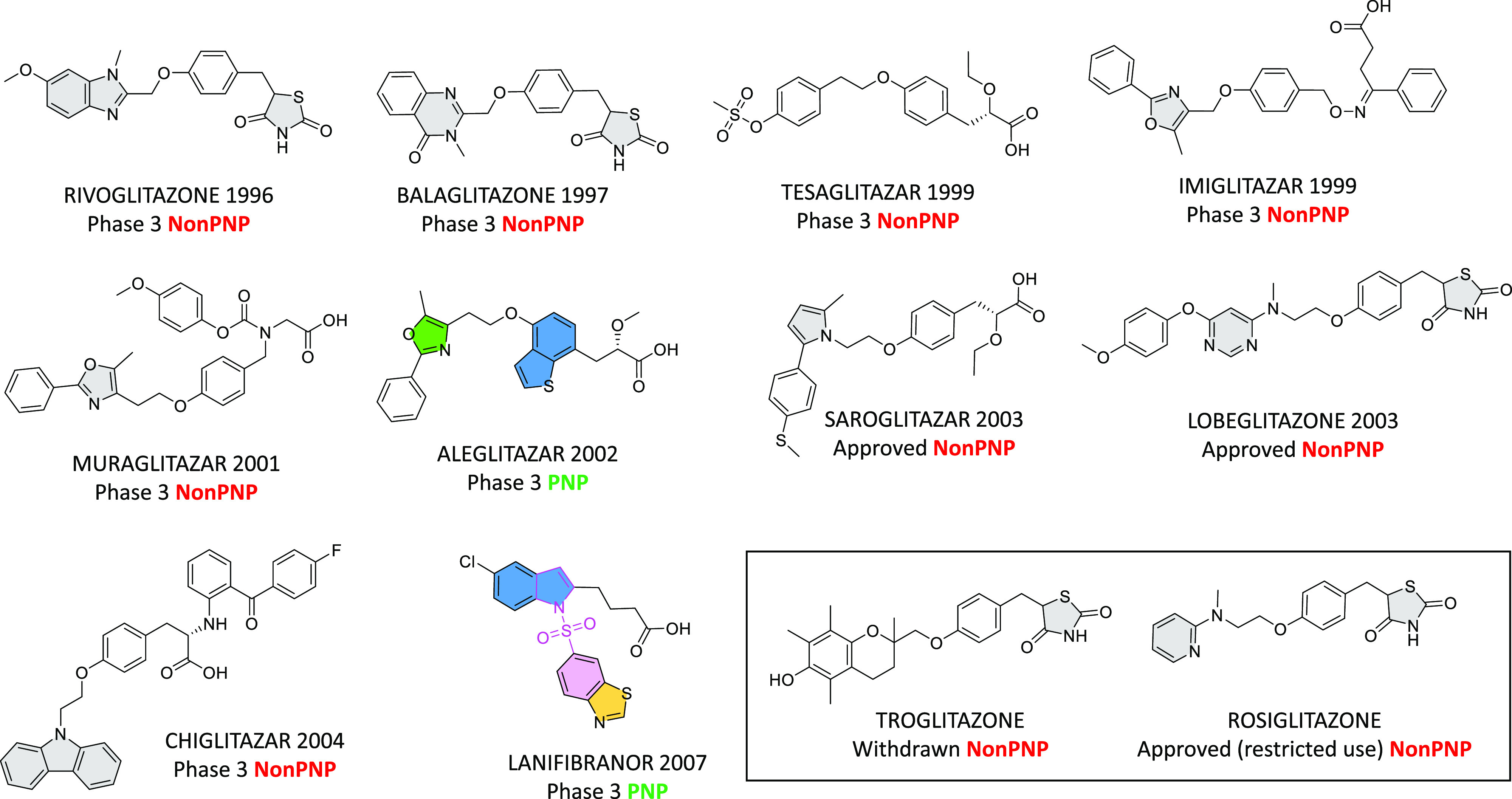
Development of Phase 3 and approved clinical PPAR agonists, where NP fragments have been infrequently used. NP fragments are filled. Color-filled NP fragments show molecules classified as PNPs. Saroglitazar (approved in India) is reported as Phase 3 in ChEMBL and lobeglitazone (approved in South Korea) was not present in ChEMBL clinical compounds. PNP occurrence in all clinical compounds active at the PPARγ subtype is 3/23 (14%) and for reference compounds, 786/3564 (22%).

The peroxisome proliferator-activated receptor (PPAR) subtypes, where PPARα and/or PPARγ agonists have been pursued for diabetes, are examples of targets that have been less responsive to the application of NP fragments. From 1990 to 2007, PNP occurrence in clinical compounds active at the PPARγ subtype (3/23, 14%) is similar to that seen in the corresponding reference compounds (786/3564, 22%). Ten PPARγ clinical compounds since 1990 have reached Phase 3, with two, saroglitizar and lobeglitazone, marketed in India and South Korea, respectively (Figure 9). These molecules are follow-ups from marketed thiazolidinones discovered before 1990, such as rosiglitazone and troglitazone (Figure 9), which suffered from cardiovascular safety issues typically encountered in this class.

## Discussion

We have used three measures of NP character to assess compounds reaching the clinic (approved drugs and phases 1–3), as well as reference compounds acting at the same biological targets. While calculated NP-likeness^27^ is in overall decline in drug discovery and clinical compounds, the results show that the “NP signal” remains strong because of the widespread exploitation of a relatively small pool of NP fragments. Notably, PNPs, which contain NP fragments but are inaccessible by Nature’s known biosynthetic pathways, have been increasing progressively over time and make up 67% of clinical compounds first disclosed since 2010. Inevitably, the growth of PNPs in clinical molecules cannot continue at the rate observed and a leveling off will occur; the 2021 year data (Figure 2a) may indicate this is underway. The overall decline in NP-likeness of clinical compounds can be attributable mainly to an increase in aromatic ring count together with a decrease in oxygen atom count.^4,36^ PNPs have introduced more ring system novelty to the clinic versus NonPNPs (Figure S2) while having lower^16^ NP-likeness (Table 4).

In support of a “natural selection” process occurring in successful drug discovery, the results (Figures 2 and 3, Table 3) show that since 2008, clinical compounds have increased NP property values versus target-matched reference compounds. Overall in this period, PNPs are 54% more likely to be found in clinical versus reference compounds. On the other hand, not all target classes show an NP signal, there is less evidence for an NP property clinical increase prior to 2008, and clinical and reference compounds have the same relative abundance in the NPL (NP-like) class in all time frames (Figure 2). In addition, the absence of a control “non-NP” fragment set means any clinical preference among the less abundant “non-NP” fragments could not be assessed.

What has caused the overall separation of PNP abundance in clinical and reference compounds published after 2008? Developments in the late 1990s to early 2000s such as the maturity of compound screening collection enhancements and multiparameter lead optimization may be contributory factors. In the same time frame, the increased attention paid to physical property control is an additional factor: higher Fsp3 and complexity, with lower carboaromatic ring count, are evident in clinical compounds versus reference compounds (Figure 4), and these trends are consistent with increased NP character.

Fourteen of the seventeen target classes show NP clinical enhancement to varying degrees, including proteases, nuclear receptors, and oxidoreductases (Table 3). In contrast, aminergic G-protein coupled receptors (GPCRs) display no clinical compound NP enhancement, while other GPCRs and protein kinases have only marginal clinical NP enhancement across the categories in Table 3. Can the diminished clinical NP enhancements in these major target classes be explained? One possible factor at play might be the historically long-established and highly exemplified options for the essential pharmacophores seen in both aminergic GPCR and kinase competitive inhibitors and the lack of selectivity commonly observed in these target classes. The majority of kinase inhibitors are competitive with ATP, and kinase drug fragments have been reported^41^ to cover a very small fraction of possible hinge-binder^42^ space. Kinase inhibitors frequently bind to multiple other kinases^43^ and existing kinase inhibitor libraries are commonly used to identify new kinase leads by focused screening.^44,45^ Competitive aminergic GPCR agonists and antagonists have historically typically contained a common aromatic ring and a basic group,^46^ as found in endogenous agonists (e.g., dopamine, serotonin, histamine, and noradrenaline), and can employ similar ligand–protein interactions within each receptor subclass.^47^ Targets among the protease, oxidoreductase, and nuclear receptor (including nuclear hormone receptors and transcription factors) families, which all show clinical compound NP enhancement, are more diverse in their substrate requirements.

Much current drug discovery uses known structures as starting points,^2,3^ and exploiting biologically proven or “privileged” structures is a prominent strategy used in developing screening collections. “Recycling” of knowledge is clearly a major factor influencing the NP profiles of clinical candidates, as shown by the target examples (Figures 7–9). Most striking is the fact that as few as 176 NP fragments account for, on average, 63% of the heavy (non-H) atoms in the Murcko scaffold structures of clinical compounds disclosed since 2008. Further, just 58 NP fragments (Figure 5) cover 90.5% of all clinical compound NP fragment space. We therefore extend the questions already raised regarding the necessity for novel ring systems in drugs^39,48^ to ask if the application of NP fragments is a preferred strategy for success, instead of hunting for diversity among the vast numbers of less-used and virtual non-NP ring systems and scaffolds.^49,50^

The rapid growth in clinical PNPs has probably occurred largely unknowingly because the PNP concept, disclosed in 2020,^12^ is too new to have had a significant impact on clinical compound design. The observed PNP and NP fragment density increases may have mostly occurred intuitively, rather than by “NP design” because the most frequently used NP fragments (Figure 5) are all very well-known to, and commonly employed by, medicinal chemists. One example of the application of an NP moiety in lead optimization is the use of *R*-(+)-carvone in the discovery of the JAK kinase inhibitor tofacitinib,^51^ itself a PNP.

The observed clinical NP fragment preferences in the data set (Figures 5 and 6) are intriguing and suggest specific fragments and combinations that could take priority in the compound design. An important point is that any specific NP fragments contained in clinical compounds, which were generally introduced at late stages in the lead optimization processes, could appear “enhanced” in clinical versus comparative reference compounds. An example is oxetane, a relatively new entry to the repertoire,^52^ which is enriched in clinical compounds (Figure 5, 8th entry in the fifth row). This effect would be less likely to be seen for NP fragments present in the starting hit structures. Quantifying the impact of these aspects is not practicable in the current dataset. Nevertheless, the narrowing of successful design options using specific NP fragments, which might occur as lead optimization progresses, is consistent with a “natural selection” hypothesis.

There is a large pool of underused NP fragments, and an immense number of novel NP fragment combinations are available to exploit to create NP-biased screening libraries and building blocks for use in lead optimization. The design principles for PNP molecules have been laid out^12−14,16,18,19^ and are ready for exploitation by medicinal chemists. PNPs provide greater ring system novelty in the clinic and predictably distinctive physicochemical properties, especially increased nitrogenous aromatic ring count versus NonPNPs. However, it is our experience that recognizing a PNP structure and knowing those NP fragment combinations that are nonbiosynthetic are often not intuitive or straightforward. Applying the available PNP algorithm^17^ in advance of synthesis is a necessary step. In designing new PNPs, synthetic challenges are likely to arise; the investment required needs to be balanced against the accumulating evidence supporting their biological relevance, clinical dominance, and untapped potential. However, we note that thousands of PNPs are now available from commercial sources, such that PNP libraries can readily be established without extensive synthesis efforts.^17^ Further extension to consider additional NP ring systems and acyclic frameworks^29,30^ is also possible.

A limitation of this study surrounds the inevitably incomplete nature of freely available reference compound datasets. To help corroborate our results, we encourage owners of complete drug discovery project databases to examine the NP properties of optimized leads and candidate drugs versus others synthesized, an approach that does not require disclosure of proprietary structures in publication.^53^ Similarly, higher NP character of identified hits versus compound screening collections, if seen, could help shed light on the presence of “dark” chemical matter.^54^ Nevertheless, the results showing clinical NP enrichment based on published compound data are sufficiently positive to warrant a greater focus on the application of NP properties and fragments in compound design.

## Conclusions

By analyzing data published in journals, extracted from ChEMBL version 32,^22^ we provide evidence that the NP properties of a set of >1000 clinical compounds published since 2008 are increased versus corresponding time- and target-matched reference compounds. The magnitude of the NP enhancement seen varies by target class, with a positive signal seen in the majority. Kinases and aminergic GPCRs, highly explored target classes with a long history and limited endogenous ligand diversity, show weak or no NP enhancement. The increase in clinical compound NP properties results from the use of just 176 NP fragments, which together make up, on average, 63% of the heavy atoms in post-2008 clinical compound core scaffolds. Clinical PNPs, where two or more NP fragments are combined in ways not achievable in nature, have been rapidly increasing over time and comprise 67% of post-2010 clinical compounds. The overall results are supportive of the occurrence of “natural selection” being associated with many successful drug discovery campaigns. It has been proposed that NP-likeness assists drug distribution by membrane transporters,^21^ and we further speculate that employing NP fragments may result in less attrition due to toxicity, a major cause of preclinical failure.^55^

There is untapped potential for further exploitation of currently used and unused NP fragments, especially in fragment combinations and the design of PNPs, without the need to resort to chemically diverse ring systems and scaffolds. To exploit these opportunities, “NP awareness” needs to be added to the repertoire of medicinal chemists. Sir James Black, discoverer of propranolol and cimetidine, famously stated that “the most fruitful basis for the discovery of a new drug is to start with an old drug.”^56^ In the genomic era, the Black principle holds ever true, as medicinal chemists, knowingly or unknowingly, are repeatedly using a small group of established NP fragments to discover clinical candidates. We concur with the sentiment that “the local chemical space of a natural product can prove superior to the natural product itself.”^57^ Adding NP fragment-based parametrization to enhance machine learning models and influence generative chemistry is recommended.

NP structural motifs are provided predesigned by nature, constructed for biological purposes as a result of 4 billion years of evolution. In short, applying nature’s building blocks—natural intelligence—to drug design can enhance the opportunities now offered by artificial intelligence.

## Supporting Information Available

NP metric clinical versus reference compounds by target class using unpaired data for all compounds and targets with ≥100 reference compounds; ring system novelty in PNPs and NonPNPs over time in clinical compounds; development of VEGF inhibitors; cross-correlation of NP measures versus other properties for post-2008 clinical compounds; and physical properties of post-2008 clinical compounds by PNP_Status (PDF)
Excel spreadsheet: NP fragment list; post-2008 clinical compound-target pairs; post-2008 Reference compounds by target; and statistical data for Figures (XLSX)
Molecular SMILES strings file (CSV)

## Abbreviations

EGFR: epidermal growth factor receptor
GPCR: G-coupled protein receptor
NK-1: neurokinin-1 receptor
NonPNP: compounds other than NP, NPL, or PNP
NP: natural product
NPL: natural product-like
PNP: pseudo natural product
PPAR: peroxisome proliferator-activated receptor
