# Supplementary material for: Occurrence of “Natural Selection” in Successful Small Molecule Drug Discovery
Source: J Med Chem. 2024 Jul 1;67(13):11226–41. doi: 10.1021/acs.jmedchem.4c00811 (PMC11247505; doi:10.1021/acs.jmedchem.4c00811)
Supplement: Supplementary file 1 — jm4c00811_si_001.pdf [file jm4c00811_si_001.pdf]

Supporting Information

Occurrence of 'Natural Selection' in Successful Small Molecule Drug Discovery

A. Lina Heinzke[1], Axel Pahl[2], Barbara Zdrazil[1], Andrew R. Leach[1], Herbert Waldmann[3,4], Robert J. Young[5], Paul D. Leeson[6,*]

1. European Molecular Biology Laboratory, European Bioinformatics Institute, Wellcome Genome Campus, Hinxton, Cambridgeshire CB10 1SD, United Kingdom
2. Compound Management and Screening Center, Max-Planck-Institute of Molecular Physiology, Otto-Hahn-Straße 11, 44227 Dortmund, Germany
3. Department of Chemical Biology, Max-Planck-Institute of Molecular Physiology, Otto-Hahn-Straße 11, 44227 Dortmund, Germany
4. Faculty of Chemistry and Chemical Biology, Technical University Dortmund, Otto-Hahn-Straße 6, 44227 Dortmund, Germany
5. Blue Burgundy Ltd, Ampthill, Bedfordshire MK45 2AD, United Kingdom
6. Paul Leeson Consulting Ltd, Nuneaton, Warwickshire CV13 6LZ, United Kingdom

* Corresponding author email: leesonpd@gmail.com

Contents

1. **Supporting Figure S1**. Post-2008 unpaired Clinical versus target Reference compound target class analysis of NP metrics, by 1) all compounds and 2) by Clinical compounds and targets with ≥ 100 Reference compounds.
2. **Supporting Figure S2.** New ring systems added to Clinical compounds by decade of first disclosure.
3. **Supporting Figure S3.** Development of VEGF2 Antagonists.
4. **Supporting Table S1.** Cross correlation R values for NP properties versus physical properties for post-2008 Clinical compounds.
5. **Supporting Table S2.** Physicochemical properties of post-2008 Clinical compounds by PNP_Status.
6. **Supporting Spreadsheet (xlsx).** Contains:
    - List of NP fragments (n=1673)
    - All Clinical compounds (n=3173) NP parameters
    - All post-2008 published Clinical compound-target pairs
    - Mean & median properties of post-2008 Reference compounds by Target
    - Statistical data for Figures 2-5, S1
7. **Molecular strings (SMILES) (csv).**

1) Unpaired, all Clinical and Reference compounds

a) % PNP

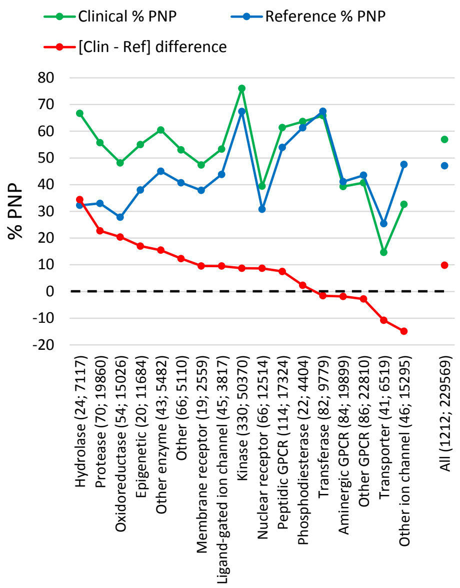

b) Frag_coverage_Murcko

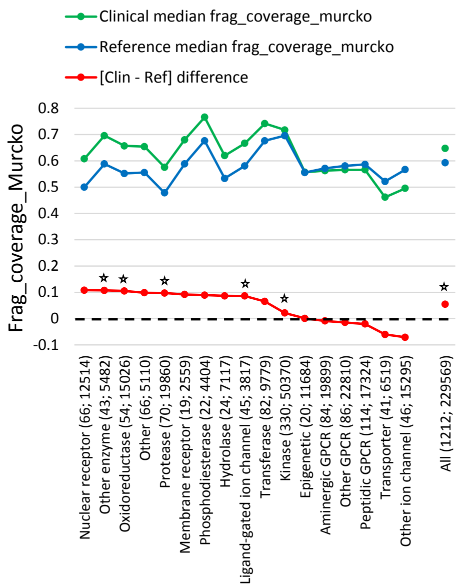

c) NP-like

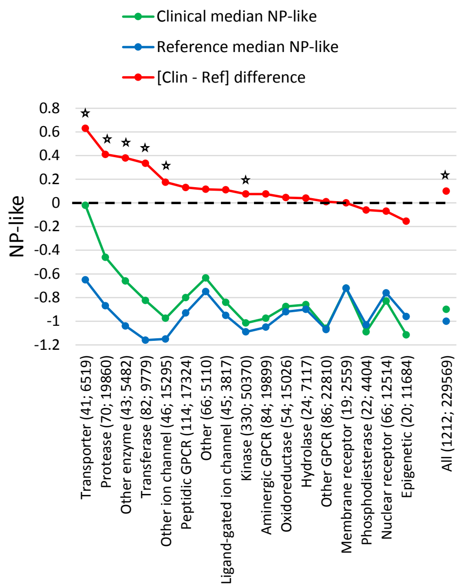

2) Unpaired, Clinical compounds and targets with ≥ 100 Reference compounds

a) % PNP

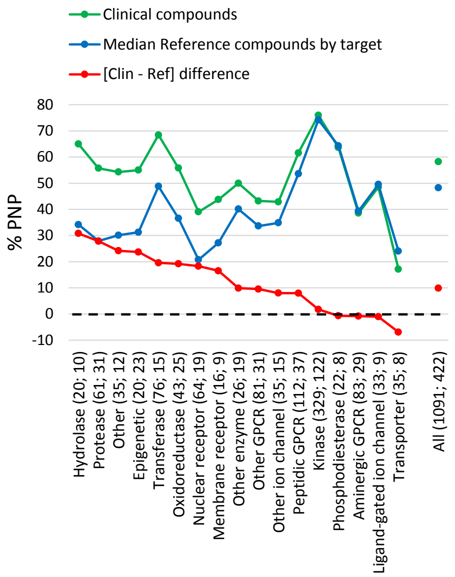

b) Frag_coverage_Murcko

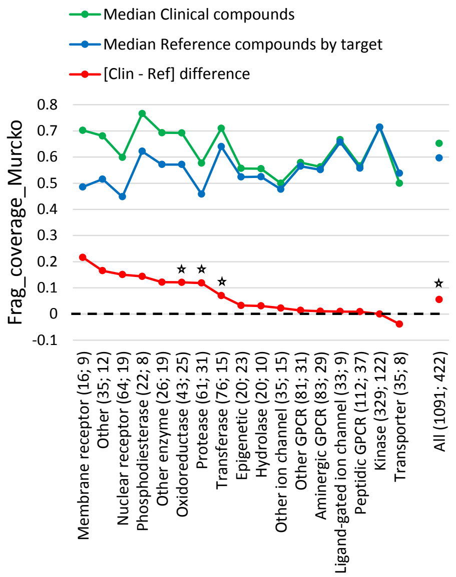

c) NP-like

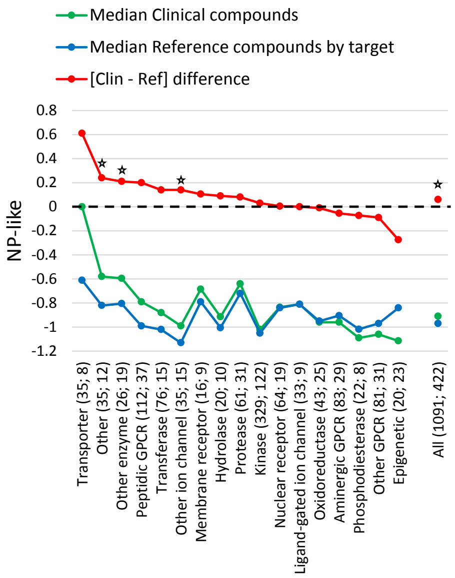

Figure S1. Post-2008 unpaired Clinical versus target Reference compound analysis of NP metrics. **1)** By all compounds. Target classes on the x-axis are shown with the numbers of Clinical and Reference compounds in parentheses. **2)** By Clinical compounds and targets with ≥ 100 Reference compounds in each target class. Target classes on the x-axis are shown with the numbers of Clinical and compounds and targets in parentheses. a) %PNP. b) Frag_coverage_Murcko. c) NP-likeness. In all cases the Clinical-Reference differences (in red) are the arithmetic differences. The black dotted lines are where Clinical and Reference values are equal. *$p < 0.05$, values from t-tests assuming unequal variances.

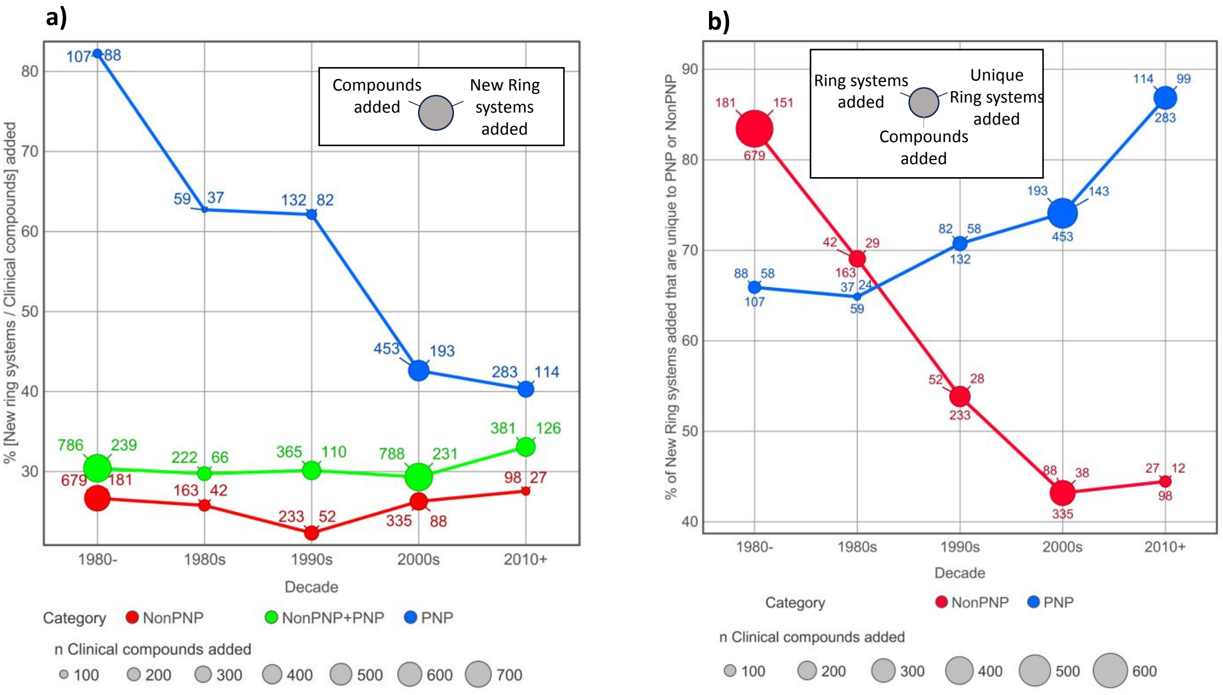

Figure S2. New ring systems added to Clinical compounds by decade of first disclosure. a) Percent new ring systems per decade by PNP, NonPNP and combined PNP+NonPNP Clinical compounds. b) Percent new rings added per decade that are unique to PNPs and NonPNPs. Ring system assignments are from DataWarrior.

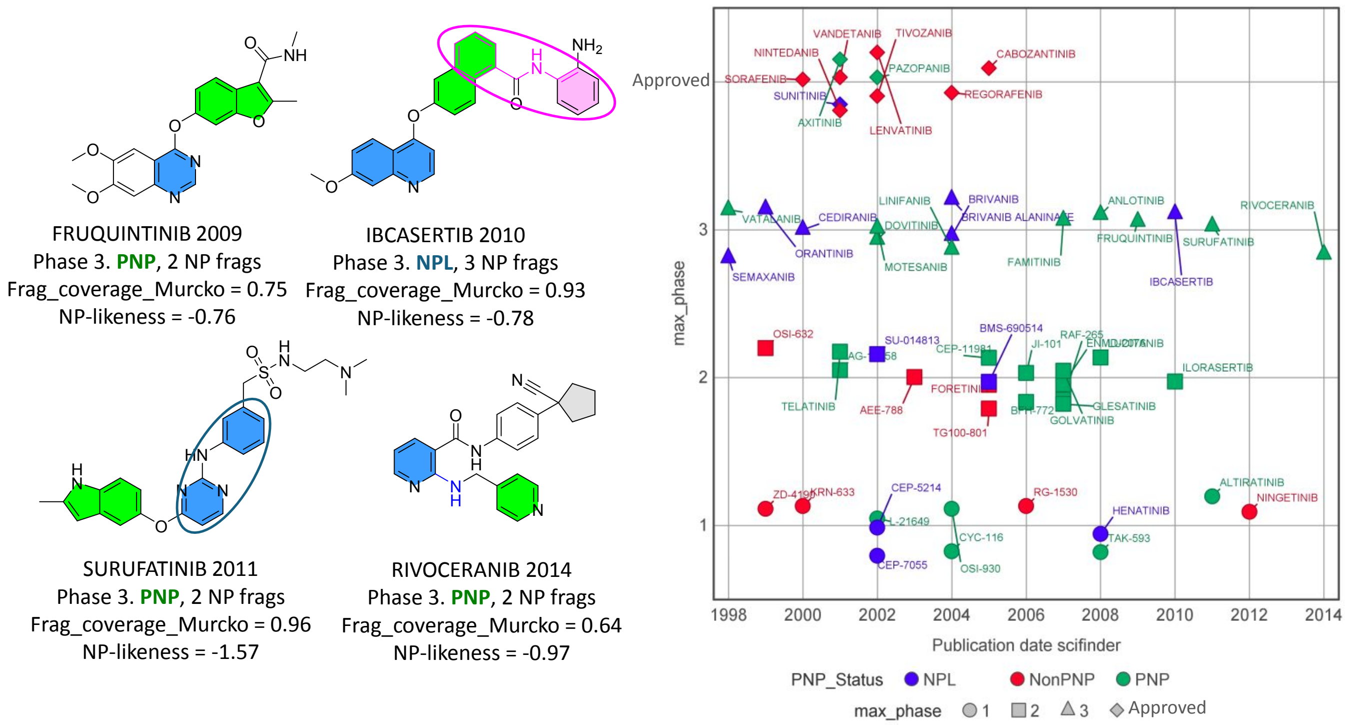

Figure S3. Development of VEGF2 Antagonists Colour filled NP fragments show molecules classified as pseudo-natural products (PNP) or NP-like (NPL). **In all**: 27 of 54 Clinical compounds are PNPs (50%) and 12 of 54 are NPLs (22%); in Reference compounds, 3566 PNP of 5886 are PNPs (61%) and 536 are NPLs (9.1%); Clinical vs Reference PNP odds ratio = 0.65 (p >0.05). **Post 2005**: 16 of 24 Clinical compounds are PNPs (67%); in Reference compounds, 1769 of 3080 are PNPs (57%); Clinical vs Reference PNP odds ratio = 1.48 (p >0.05). **Clinical compounds only**: pre vs post 2005 PNP versus others odds ratio = 3.45 (p = 0.0312).

Table S1. Cross correlation R values for NP properties versus physical properties for post-2008 Clinical compounds having ≥ 100 reference compounds acting at their targets (n=1091 except where shown).

Property	R Values[a]		
	PNP	Frag_coverage _murcko	NP_Like
**frag_coverage_murcko**	**0.528**		
**NP_Like**	-0.159	0.001	
**MW**	0.177	-0.055	0.099
**ALogP**	0.044	-0.123	-0.031
**cx_LogP**	-0.025	-0.150	-0.070
**cx_LogD**	0.034	-0.109	-0.097
**HBA**	0.286	0.135	-0.001
**HBD**	-0.068	-0.041	0.247
**PSA**	0.123	0.044	0.139
**RotB**	-0.107	-0.160	0.071
**HA**	0.203	-0.025	0.111
**QED**	-0.096	0.069	-0.070
**Fsp3**	-0.021	0.066	0.448
**n Carboaliphatic rings**	0.126	0.124	0.405
**n Heteroaliphatic rings**	0.153	0.072	0.210
**n Carboaromatic rings**	-0.165	-0.287	-0.094
**n Heteroaromatic rings**	**0.493**	0.378	-0.381
**n Stereocenters**	-0.063	0.003	**0.637**
**nSPS**	0.056	0.143	**0.606**
**pChEMBL** (n=720)	0.108	-0.005	-0.012
**LE** (n=717)	-0.128	0.090	0.022
**LLE** (n=717)	0.041	0.138	0.038

[a]PNP values are categorical, i.e. 1 or 0. The high NP fragment and heteroaromatic ring count seen in PNPs (Table S2) are reflected by the highest R values. NP-like shows the strongest correlations with stereocenter count and the spacial complexity score (nSPS).

Table S2. Physicochemical properties of post-2008 Clinical compounds by PNP_Status. p Values are from unpaired t-tests.

Property	PNP_Status	n	Mean	Median	Std. Dev.	p vs PNP
MW	PNP	666	477	458	119	
	NonPNP	368	437	436	110	0
	NPL	123	447	431	132	0.023
ALogP	PNP	666	3.8	3.8	1.7	
	NonPNP	363	3.8	3.7	1.8	NS
	NPL	122	3.1	3.4	2.2	0.002
cx_LogD	PNP	666	2.5	2.7	2.1	
	NonPNP	363	2.5	2.5	2.0	NS
	NPL	122	1.7	1.9	2.8	0.002
HBD	PNP	666	1.79	2	1.13	
	NonPNP	363	1.99	2	1.27	0.014
	NPL	122	2.10	2	2.02	NS
HBA	PNP	666	6.79	7	2.17	
	NonPNP	363	5.36	5	2.19	0
	NPL	122	6.08	5	3.26	0.023
PSA	PNP	666	98.72	95.3	34.6	
	NonPNP	363	87.83	88.4	35.6	0
	NPL	122	99.30	87.0	57.6	NS
Fsp3	PNP	666	0.35	0.35	0.17	
	NonPNP	368	0.36	0.33	0.18	NS
	NPL	123	0.41	0.38	0.20	0.002
n Carboaromatic rings	PNP	666	1.35	1	0.89	
	NonPNP	368	1.71	2	0.86	0
	NPL	123	1.33	1	0.85	NS
n Heteroaromatic rings	PNP	666	1.86	2	1.01	
	NonPNP	368	0.73	1	0.72	0
	NPL	123	1.12	1	0.92	0
n Aromatic N atoms	PNP	666	2.80	3	1.72	
	NonPNP	368	1.13	1	1.32	0
	NPL	123	1.53	1	1.42	0
n Carboaliphatic rings	PNP	666	0.47	0	0.85	
	NonPNP	368	0.24	0	0.77	0.00001
	NPL	123	0.39	0	0.82	NS
n Heteroaliphatic rings	PNP	666	1.06	1	1.02	
	NonPNP	368	0.66	1	0.77	0
	NPL	123	1.12	1	0.90	NS
n Stereo centres	PNP	666	1.17	1	1.90	
	NonPNP	368	1.23	1	1.86	NS
	NPL	123	2.33	1	2.74	0.00001
RotB	PNP	666	5.80	5	2.74	
	NonPNP	363	6.93	6	3.79	0
	NPL	122	5.93	6	3.58	NS
QED	PNP	666	0.49	0.48	0.19	

	NonPNP	363	0.51	0.52	0.21	NS
	NPL	122	0.52	0.51	0.23	NS
**nSPS**	PNP	666	17.9	16.7	6.30	
	NonPNP	368	16.3	14.6	6.23	0.00008
	NPL	123	20.9	18.8	8.06	0.00019
**n NP frags**	PNP	666	3.17	3	1.01	
	NonPNP	368	1.22	1	0.80	0
	NPL	123	2.40	2	0.62	0
**frag_coverage_murcko**	PNP	666	0.74	0.75	0.18	
	NonPNP	368	0.38	0.38	0.24	0
	NPL	123	0.68	0.67	0.20	0.00171
**NP-like**	PNP	666	-0.89	-0.97	0.65	
	NonPNP	368	-0.70	-0.85	0.72	0.00002
	NPL	123	-0.39	-0.55	0.94	0
**pChEMBL**	PNP	424	8.0	8.2	1.2	
	NonPNP	213	7.8	7.9	1.2	NS
	NPL	59	7.9	7.9	1.0	NS
**LE**	PNP	424	0.34	0.34	0.082	
	NonPNP	212	0.36	0.36	0.087	0.004
	NPL	58	0.36	0.36	0.096	NS
**LLE**	PNP	424	4.3	4.4	1.8	
	NonPNP	212	4.0	4.1	1.8	NS
	NPL	58	4.6	4.8	2.0	NS
